# Can neural networks model the human perception of geometric shapes?

**DOI:** 10.1371/journal.pcbi.1014554

**Published:** 2026-07-27

**Authors:** Maxence Pajot, Théo Morfoisse, Mathias Sablé-Meyer, Yair Lakretz, Stanislas Dehaene

**Affiliations:** 1 Cognitive Neuroimaging Unit, CEA, INSERM, Université Paris-Saclay, NeuroSpin center, Gif/Yvette, France; 2 Sainsbury Wellcome Centre for Neural Circuits and Behaviour, University College London, London, United Kingdom; 3 Laboratoire de Sciences Cognitives et Psycholinguistique, ENS, Paris, France; 4 Collège de France, Université Paris Sciences Lettres (PSL), Paris, France; Basque Center on Cognition Brain and Language, SPAIN

## Abstract

Artificial neural networks achieve impressive success in many vision tasks. Nevertheless, previous work has suggested that their representation of geometric shapes only partially captures the representations found in humans, who additionally require a symbolic “language of geometry”. In this study, we evaluate the progress in this area by systematically comparing human performance in three geometric shape perception tests, with the predictions of recent Convolutional Neural Networks (CNNs) and Vision Transformers, varying in size, training datasets and training methods, to recognize and process geometric shapes and compare these representations to the ones found in humans. In two tasks probing quadrilateral and geometric shape perception, introduced by Sable-Meyer et al. (2022, 2026), recent neural networks exhibit representations similar to humans and predict human performance better than a symbolic model. In a third task, involving geometric drawings, the networks still fell short of human performance. Across the three experiments, the main factor driving similarity between human and neural network representations was the size of the training dataset, rather than a specific architecture or the number of parameters of the model. Together, our results show that a massive scaling-up of the training data helps neural networks approximate human-like representations of geometric shapes, but that they still fall short of fully capturing the human representations of geometric shape.

## Author summary

Recent neural networks have achieved remarkable success across a wide range of visual tasks, often rivaling or surpassing human performance. Yet, it remains unclear whether these systems truly reflect the way humans perceive and reason about the world. In this study, we addressed this question in the domain of geometry—a fundamental aspect of human visual understanding. We compared human performance with that of modern vision models across three tasks involving geometric shapes and drawings. The latest networks captured many features of human perception and even outperformed symbolic models previously thought to better reflect human reasoning. However, they still fell short of fully explaining human behavior, especially in recognizing geometric drawings. The main factor behind their human-like behavior was the scale of their training data, not their architecture or number of parameters. These findings highlight both the progress and the remaining gap between deep learning and human geometric intuition.

## 1. Introduction

**Fig 1:**
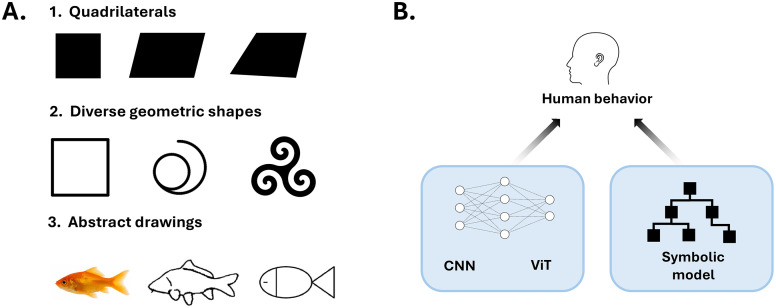
Geometric stimuli and modeling framework to evaluate geometric shape perception in neural networks. **A**. Schematic depiction of the 3 different geometric tasks on which the neural networks were tested: 1, detection of outliers within quadrilateral shapes; 2, memory for geometry signs; 3, matching of geometric drawings with photos. **B.** In each case, we tested how different neural networks can model human behavior, and how they compare to symbolic models of shape perception.

The ability to understand and generate geometric shapes has often been considered a distinctive feature of human cognition [1–3]. Here, we use this term “geometric shape” to refer both to non-figurative shapes that consist of simple lines and curves organized by spatial regularities, and to schematic figurative drawings depicting objects or scenes using such geometric elements (see Fig 1 for examples). While studies on non-human primates [3,4] and birds [5], have demonstrated a capacity for abstract visual information processing, there is some preliminary evidence that these species recognize, generate, and manipulate geometric shapes in a way that is quite distinct from humans. Indeed, even after extensive training, such behaviors have not been observed in non-human animals, whereas human children, from a very early age, appear to engage with abstract figures effortlessly, even without explicit instruction [4,6,7]. Recent work by Schmidbauer, Hahn, and Nieder [8] has added nuance to this picture by suggesting that crows may be capable of using some abstract geometrical features, but this finding was criticized [9] for using only a very small number of shapes and large differences that could be processed non-geometrically. In humans, the ability to perceive and produce abstract geometric regularities is observed across diverse cultural contexts, including populations with little formal education, such as indigenous groups in Namibia and the Amazon [3,10,11], suggesting that it is not solely a product of Western schooling. Archaeological evidence further indicates that early Homo sapiens engaged in geometric mark-making at least 73,000 years ago [12], and even earlier engravings, dating back 540,000 years, have been attributed to Homo erectus [13].

In line with the language of thought hypothesis [14], Sablé-Meyer et al. [15] suggested that humans encode geometric features symbolically. By symbolic, we refer to the representational format posited by the language of thought hypothesis: systems composed of discrete elements that can be composed into complex expressions according to a set of explicit rules, a grammar [1]. This proposal extends the language of thought hypothesis to geometric perception, treating shape cognition as one of several uniquely human abilities, alongside natural language, mathematics, and music, that may rest on a shared capacity for recursive, compositional representation [16,17]. The mental representation of a shape would be encoded as a “mental program” in this internal language of geometry: a compositional expression that draws the shape by combining a small set of primitives according to grammatical composition rules. Their research demonstrated that a model incorporating symbolic representations of an object’s features (e.g., parallelism, or right angle) provides a better account of human behavior than standard vision models [3], including those with a high “brain score” such as CorNet [18].

While the symbolic model offers a compelling framework for understanding the types of computations the brain might perform when processing geometric shapes, how the brain encodes symbols and rules remains unclear. Recent advancements in artificial intelligence have rekindled the debate between symbolic and connectionist theories of cognition [19,20], with some arguing that the success of large neural networks challenges the usefulness of symbolic theories [21,22], while others maintain that symbolic descriptions remain indispensable [1,23]. In contrast to symbolic systems, a connectionist model processes information through distributed patterns of activation across a network of simple units. Rather than relying on discrete representations and explicit rules, it learns continuous-value connection weights to capture complex patterns, relying only on the constraints of its architecture and training. Connectionist models have the advantage of relying on mechanisms that may be closer to those implemented in the brain [24]. However, this advantage is meaningful only if these models also reproduce the behavioral patterns observed in humans. For geometric shape perception, initial findings, such as those by Sablé-Meyer et al., suggested a significant divergence, indicating that convolutional neural networks (CNNs) bear a closer resemblance to non-human primate visual processing. This view was reinforced by subsequent work [25] that criticized the importance put on deep neural network as models of human perception. However, more recent research by Campbell et al. [26] suggested that more advanced large-scale models, with billions of parameters and very large training sets, exhibit performance that resembles that of humans.

In the present work, we ask whether modern neural networks can capture human geometric shape perception, and if so, what makes them able to do so. We do not try to adjudicate between symbolic and connectionist accounts of geometric cognition, but rather pursue a more modest and tractable goal: characterizing the conditions under which neural networks representations align with humans on geometric tasks. Our contributions are two-fold: first, we reproduce and extend results from Campbell et al., [26] to a broader set of tasks and networks. Second, we examine which factors drive human-like performance, such as model architecture (e.g., CNNs vs. Transformers), model size (number of parameters), the size of the training data, or the training method involved (contrastive learning, supervised, self-supervised).

To that end, we looked at how closely neural networks align with human behavior on three different tasks with varying levels of difficulty (Fig 1A). In the first task, we looked at how they represent quadrilaterals with different levels of geometric regularity. We used Representational Similarity Analysis (RSA) to compare the internal representations of humans (as estimated from their behavioral responses) and of neural networks on quadrilaterals. In the second one, we compared neural networks to human data on a self-paced delayed-match-to-sample task: after seeing a shape for as long as needed, participants were asked to remember it through a delay and find it among 6 possible choices. In the third task, we examined how neural networks represents a dataset of images, depicting 24 entities (e.g., faces or objects) rendered in 3 different formats varying in their level of abstraction: color photos, line drawings, and geometrical compositions (e.g., a fish depicted with an ellipse and a triangle). Together, these three tasks probe the ability of neural networks to capture human geometric cognition across increasing levels of complexity. Experiment 1 examines shape representations in a narrowly controlled setting, the perception of quadrilaterals; Experiment 2 extends this to a broader repertoire of non-figurative geometric shapes. Experiment 3 turns to the perception of abstract figurative drawings, another hallmark of human shape cognition.

We tested two widely used vision architectures: Convolutional Neural Networks (CNNs) and Vision Transformers (ViTs). CNNs, initially inspired by biological vision [27], have been studied as models of visual cognition due to their correlation with neural activations in the primate visual cortex [28,29]. However, they have faced criticism as models of human perception [25,30], in particular for relying more heavily on texture than on shape [31] and for exploiting surface regularities in their training data [32]. In the domain of geometric tasks, earlier CNNs captured non-human primate perception but failed to reproduce the human sense of geometric regularity [3]. ViTs, by contrast, are a more recent architecture that leverages the self-attention mechanisms of the transformer model [33], allowing them to capture global relationships within an image without the inductive biases of CNNs. They have been shown to rely more on shape than on texture, aligning more closely with human visual processing [34,35].

As a baseline, we compared these neural networks to the symbolic models introduced by [3,15]. These models serve as an established benchmark that previously captured human behavior better than earlier neural networks, thus providing a meaningful point of comparison for assessing whether modern neural networks now match or exceed symbolic accounts of geometric cognition.

In the first task, we found that the best contemporary neural networks can achieve representations of quadrilaterals that are very close to humans, bearing closer resemblance to human behavior than the symbolic model introduced by [3]. In the second task, on a more diverse set of geometric shapes, neural networks also explained human behavior better than a symbolic model. However, in the third experiment, while achieving impressive results, neural networks still fell short of human level. In all experiments, we found that the size of the training dataset is a crucial factor for alignment with human behavior.

## 2. Results

### 2.1 Experiment 1. Can neural networks capture the geometric regularities of quadrilaterals?

**Fig 2:**
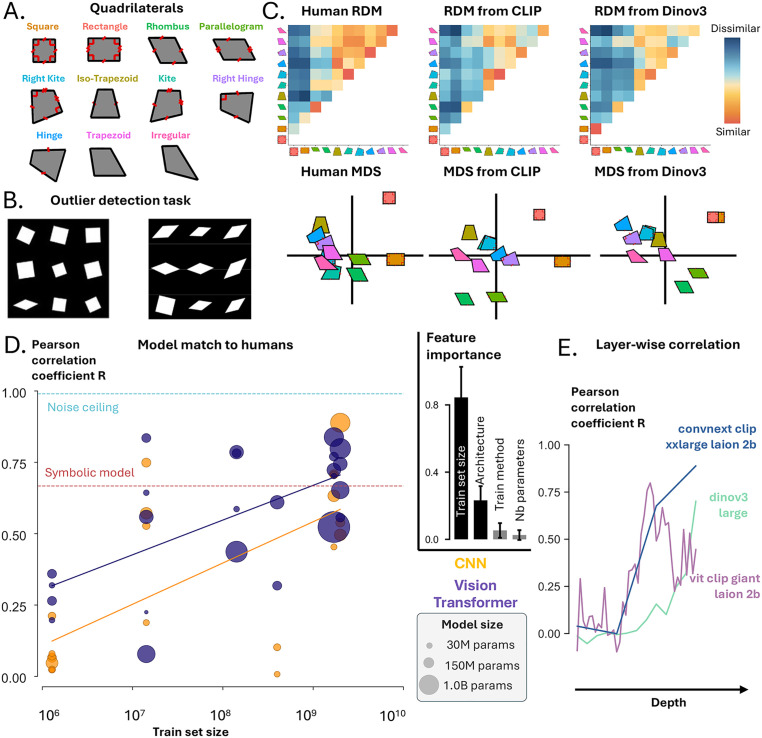
Representation of quadrilaterals of varying geometric regularity in different neural networks. **A.** The eleven different quadrilaterals tested, ordered by geometric regularity from left to right and top to bottom. **B.** Two example trials of an outlier detection task. **C.** Representation Dissimilarity Matrices (RDMs) for humans and two neural networks (CLIP and Dinov3). Very regular quadrilaterals (square, rectangle) are very similar to one another, while being very dissimilar to irregular quadrilaterals. Projection in 2D using Multi-Dimensional Scaling (MDS) of the above RDMs. The arbitrary dimensions of the MDS are aligned between networks for ease of reading. **D.** Correlation between the RDMs of the neural networks and the behavioral RDM, plotted as a function of training set size. Color indicates architecture, and dot size the number of parameters of the model. The best models have a higher correlation with human behavior than the symbolic model from Sablé-Meyer et al. [3]. Inset: Feature importance analysis. Size of the training dataset appears as the most important predictor of the alignment of a model with human behavior. **E.** Correlation of the RDMs taken at each layer of three high-performing networks. Correlation is highest within the deeper layers.

We first investigated how neural networks represent 2D quadrilateral shapes (Fig 2A). We leveraged behavioral data from Sablé-Meyer et al. [9], who conducted an outlier detection task with human participants (Fig 2B). From this, a Representational Dissimilarity Matrix (RDM) was derived using participants’ accuracy and response time [36]. This matrix quantified human perceptual dissimilarity between pairs of quadrilaterals (greater dissimilarity corresponded to easier outlier detection).

To compare human perception with neural networks representations of shapes, we also computed RDMs for neural networks. For each pair of quadrilaterals, we computed the Euclidean distance between different combinations of scales and rotations of the quadrilaterals in the network’s embedding space, then averaged these distances to obtain a measure of dissimilarity between these two shapes. By comparing neural network RDMs with the behavioral RDM, we obtained a measure of alignment between the model and human representations. We also computed the dissimilarity with other metrics (cosine similarity and correlation). All results were reproduced (S2 Fig), and in all subsequent analyses, unless mentioned otherwise, we only consider the Euclidean distance. The RDM can be computed at every layer of the networks. Where previous work has mainly focused on embeddings at the last layer [3, 9], we chose the layer that showed the best correlation with human behavior. In close to half of the networks (47%), the best layer was the last one; for the others, the best layer was in the range of 50% depth (middle layer) to 95% depth (penultimate layer).

As a baseline, we correlated the human RDM with a dissimilarity matrix from a symbolic model proposed by Sablé-Meyer et al. [3]. This model represents quadrilaterals using their inherent geometric properties such as right angles or the presence of parallel lines. For each shape, a property vector could then be computed as the vector of the properties possessed by the quadrilateral. We then computed the dissimilarity of two shapes for the symbolic model as the L1 distance between the corresponding property vectors.

Sable- Meyer et al. [9] had observed that human dissimilarity was mainly driven by the geometric regularity of the shape: number of parallel lines, right angles, sides with equal lengths. Fig 2C shows that the square and rectangle are very dissimilar to all other less regular quadrilaterals. Quadrilaterals of intermediate regularity, such as the parallelogram and the rhombus, stand between the two.

Qualitatively, Fig 2C show that some networks had representations quite similar to human – here, two Vision Transformers, trained on LAION-2B (CLIP) and on LVD-1697M (Dinov3). To quantify this, we computed the correlation between the RDMs from the models and the behavioral RDM. We saw that the best models achieved a correlation of close to 0.9 with the human RDM, much higher than the correlation of 0.68 of the symbolic model. However, these correlations remained below the empirical noise ceiling of 0.99, estimated by computing the split-half reliability of participants’ behavioral RDMs. We used Pearson correlation; however, Pearson and Spearman correlation results were highly consistent with each other (S3 Fig).

Could the models tested, even with high correlation to human behavior, rely on low-level visual features of the shapes, and not on their geometric properties? In Sablé-Meyer et al., [9] the authors had found that a linear model with RDMs from both the symbolic model and a CNN, thought to be a good model of the primate visual system [18], could accurately model human behavior, with the symbolic model having the greatest contribution. They suggested that even though geometric properties were the most important factor, humans also relied on low-level visual information. In a similar analysis, we used the same predictors as Sablé-Meyer et al., (2025), and added the RDM of a model of the Dinov3 family, with high correlation with human behavior. In that case, we found that most of predictive power comes from Dinov3 (β = 0.107, p < 10^-8^), and that the symbolic model is not a useful addition when predicting human dissimilarities (β = 0.005, p = 0.726), while the tested CNN proved to be barely significant (β = -0.020, p = 0.042). This result suggests that the best models fully capture human behavior by relying on both the geometric properties of the quadrilaterals as well as on lower-level visual features.

To better understand which factors made this possible, we systematically evaluated various vision models, including Vision Transformers [33] and Convolutional Neural Networks (CNNs), to identify architectural and training factors critical for developing human-like abstract shape perception. These models differed in their training methods: classification, unsupervised [37] and contrastive learning [38]. The models also varied in the number of parameters (from 20 millions to 7 billion), and in the size of the training dataset, the smallest being Imagenet-1k [39] and the largest being LAION-2B [40]. Characteristics of all the different models we tested can be found in supplementary material.

In Fig 2D, we plotted the correlation between each network’s predictions and human behavior as a function of training dataset size. Each point represents a model, with point size indicating the number of parameters and color denoting architecture (CNN vs. Transformer). A clear trend emerged: the primary determinant of alignment with human responses appeared to be the amount of training data. All models trained on LAION-2B—the largest dataset in our sample—achieved substantially higher correlations with human behavior than the symbolic model. Conversely, models trained on ImageNet-1k, the smallest dataset considered, consistently underperformed relative to the symbolic baseline.

To disentangle the contributions of various model attributes, we applied ridge regression to predict the correlation with human data using four factors: dataset size, parameter count, architecture type (an indicator variable equal to 1 when the network is a Vision Transformer, and 0 if it is a CNN) and training method (an indicator variable for each of the training methods: classification, contrastive learning and unsupervised). We then used permutation feature importance analysis: we measured the drop in performance of our regression when shuffling the values of a specific variable, or set of variables, showing its contribution to the final output. This analysis revealed that dataset size is the dominant predictor of behavioral alignment (Fig 2D), with an average drop ${\Delta R}^{\text{2}}=\text{0}.\text{8}\text{4}\;\pm \text{0}.\text{1}\text{8}$ (mean ± SD across 30 permutations). Vision Transformers showed a modest advantage over CNNs (${\Delta R}^{\text{2}}=\text{0}.\text{2}\text{3}\;\pm \text{0}.\text{0}\text{8}$), whereas training method and model size, indexed by number of parameters, had no measurable impact (${\Delta R}^{\text{2}}=\text{0}.\text{0}\text{5}\;\pm \text{0}.\text{0}\text{4}$ and ${\Delta R}^{\text{2}}=\text{0}.\text{0}\text{3}\;\pm \text{0}.\text{0}\text{3}$ respectively). The non-overlapping distributions indicate that dataset size contributes substantially more than any other factor. A feature was deemed significant if its mean drop exceeded the Bonferroni-adjusted z-threshold. To further verify that the findings were not driven by systematic differences between architectures or training pipelines, we complemented the regression analysis with a linear mixed-effects model including random intercepts for training method and backbone architecture. This analysis confirmed a robust positive association between training dataset size and behavioral alignment (β = 0.074, p = 0.001), whereas the number of parameters had no significant effect (p = 0.26). Allowing the slope of dataset size to vary across training methods did not improve model fit (p = 0.96). The relationship between dataset size and behavioral alignment is therefore stable across training schemes.

Several caveats must be considered when interpreting these findings. First, in the models analyzed, the number of parameters is moderately correlated with the size of the training dataset (Pearson correlation coefficient R = 0.32), making it challenging to fully disentangle their respective contributions. This confound arises from a practical constraint: large-scale networks are rarely trained on small datasets, just as small networks are seldom trained on vast amounts of data. A second issue concerns model architecture. Vision Transformers, being more recent, have demonstrated superior scalability with large datasets compared to classical convolutional neural networks (CNNs). Nonetheless, not all CNNs included in our analysis are outdated; for instance, we evaluated ConvNeXt models, a modern CNN architecture explicitly designed to scale in a manner comparable to Vision Transformers [41]. When restricting the feature importance analysis to Vision Transformers and ConvNeXt models, architectural differences no longer significantly predicted performance (p = 0.17).

At what depth within a neural network do representations become most abstract and best aligned with human behavior? While our main analyses focused on each model’s best-performing layer, it is informative to examine alignment across all layers. In Fig 2E, we presented the correlation with human behavior at each layer of three networks. Interestingly, while various architectures, sizes, and training regimes could yield models that aligned well with human data overall, the specific layer at which this alignment peaked varied markedly. For all convolutional neural networks tested, the highest alignment with human behavior consistently occurred in the final layer, whereas it was not the case for all Vision Transformers. For ViTs trained on image/text pairs, the strongest alignment emerged in the middle layers, a pattern that parallels findings in large language models, where abstract linguistic features such as syntax are also most robustly encoded in intermediate layers [42,43]. S2 Table provides a complete overview of all models and their best-aligned layer for each experiment.

### 2.2 Experiment 2. Can neural networks capture the Language of Thought for various geometric shapes?

**Fig 3:**
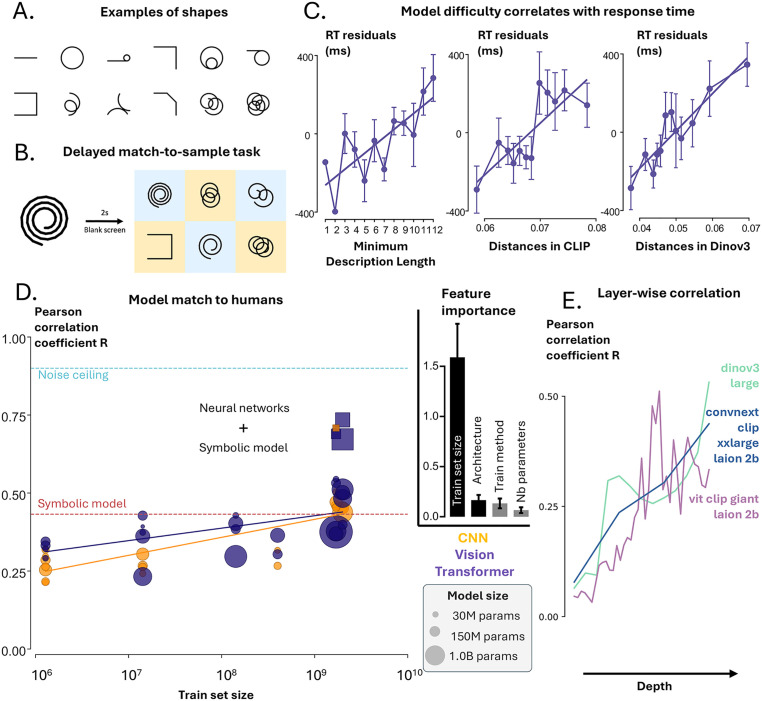
Representation of geometric shapes of varying geometric regularity in different neural networks. **A.** Example of twelve of the generated shapes, with one shape per cost, from 1 (top left) to 12 (bottom right). **B.** Example of a trial in a delayed match to sample task: after a delay, the participant must find, within the six proposed choices, the one that matches the sample shape. **C.** Regression on the residuals of the reaction time after regressing out the luminance of the image. As in Sablé-Meyer et al., [15], we see that cost (or MDL) is positively correlated with the response time. In both CLIP and Dinov3, the sum of inverse distances is positively correlated with the residual response time. Error bars represent the standard deviation of residual choice time, across shapes in the same bin. **D.** Correlation between trial difficulty for neural networks and trial difficulty for humans, plotted as a function of training set size. Color indicates architecture, and dot size the number of parameters of the model. The best models have a higher correlation with human behavior than the symbolic model from Sablé-Meyer et al. (2022). Squares represent the R values for the five best models when adding the MDL: adding information from the symbolic model does improve alignment, even for the best models. Inset: Feature importance analysis. Size of the training dataset appears as the most important predictor of the alignment of a model with human behavior. **E.** Correlation with human behavior at each layer of three high-performing networks. Correlation is highest within the deeper layers.

In the preceding section, we showed that artificial neural networks could develop shape representations that aligned with human perception. This conclusion, however, was based on a restricted set of 11 quadrilaterals. To broaden the scope of analysis, we now examine a more diverse set of 68 geometric shapes introduced by Sablé-Meyer et al., [15] (Fig 3A). These stimuli were generated using a formal language that produces line drawings via recursive compositions of simple geometric primitives. Each shape is associated with a corresponding program, and its complexity is quantified by the length of the shortest program capable of generating it—its Minimum Description Length (MDL).

To compare human and model representations, we relied on behavioral data from the same study. Participants were engaged in a self-paced delayed match-to-sample (DMTS) task in which they could see a target shape for as long as needed (encoding time), followed by a brief delay and then identification among five other distractors (Fig 3B). Both encoding time and choice time were found to increase with MDL, indicating that more complex shapes—those with higher MDL—were harder to memorize and recognize.

In this experiment, we could not apply representational similarity analysis (RSA) as in the previous section, due to the absence of a well-defined measure of dissimilarity between shapes. Indeed, in the earlier quadrilateral task, each trial involved a direct comparison between two shapes, allowing for straightforward dissimilarity estimation. Here, however, participants were asked to identify a target shape among five distractors. While inverse response time could, in principle, be modeled as the sum of dissimilarities between the target and each distractor [44,45], this approach was not applicable in the present context because the distractors were not selected randomly from the full stimulus set; instead, two were chosen to be among the closest to the target in terms of luminance, and two others were selected based on proximity in the CorNet embedding space [46], as described in [15]. As a result, the construction of a reliable representational dissimilarity matrix (RDM) was not feasible in this case.

In the absence of a reliable dissimilarity matrix, we instead compared the relative difficulty of individual trials as experienced by humans and neural networks. For human participants, given the low error rates, trial difficulty was indexed by the choice time—the duration required to spot the target shape among distractors [The time taken to encode the shape could, in theory, be modeled as well. However, this paper focuses on representational geometries using measures of dissimilarities between shapes. Encoding time is dependent on the difficulty of representing a single shape, no matter the others, and we thus chose not to pursue this direction.].

For neural networks, we estimated the difficulty of each trial based on the visual similarity between the target and the five distractors. Difficulty was quantified using a measure of cumulative distraction which, on each trial, was based on the sum of the inverse distances between the target shape and each of the distractors (see Methods for a detailed description).

As stated above, we were interested in studying the correlation between the difficulty of trials for humans and for neural networks. However, there is a confounding factor present here: the luminance of the images presented. The more complex a shape is in the language of thought, the more lines are used on average, and thus the darker the overall image. Then, a simple model that captures only the luminance of an image would be a good predictor of the difficulty. To avoid this confound, in all subsequent analyses, we first regressed out the luminance of the target image on the choice time. We also ran an additional analysis controlling for more confounds (e.g., spatial frequency, number of intersections) and the results remain unchanged (S3 Fig).

We first reproduced the previous result by Sablé-Meyer et al., [15]. Fig 3C shows that, even after regressing the luminance of the reference image to the choice time, the MDL of the shape is a good predictor of the difficulty: the higher the MDL, the more difficult the trial.

For both networks tested (CLIP and Dinov3), we found that the sum of inverse distance (i.e., the cumulative distraction) closely mirrored the difficulty observed in human performance, suggesting that these neural networks encode geometric shapes similarly to humans.

Building on our previous analysis, we further investigated which factors enabled neural networks to develop abstract shape representations. As Fig 3D illustrates, a consistent trend emerged: only models trained on the largest datasets (LVD-1697M and LAION-2B) achieved better correlation with human behavior than the symbolic model. To understand the specific contributions of various model attributes, we again conducted a regression analysis. This allowed us to predict each network’s performance based on its number of parameters, training dataset size, architecture type, and training paradigm. Consistent with our earlier findings, permutation feature importance analysis (Fig 3D) once more showed that training dataset size was the dominant factor driving the alignment of neural networks with human behavior (${\Delta R}^{\text{2}}=\text{1}.\text{5}\text{5}\;\pm \text{0}.\text{2}\text{0}$). Architecture was again a significant predictor when looking at all the networks (${\Delta R}^{\text{2}}=\text{0}.\text{1}\text{7}\;\pm \text{0}.\text{0}\text{2}$); when limiting the tested CNNs to those of the ConvNext family, we once again found that the effect of architecture became non-significant. As in Experiment 1, we complemented this regression analysis with a linear mixed-effects model including random intercepts for training method and backbone architecture. This analysis again revealed a positive relationship between training dataset size and behavioral alignment (β = 0.024, p < 10 ⁻ ⁸). In contrast, the number of parameters did not reliably predict performance (p = 0.051). Allowing the slope of dataset size to vary across training methods did not improve model fit (p = 1), indicating that the relationship between dataset size and alignment is consistent across training schemes.

In summary, both symbolic model and neural networks could partly predict human behavior. Was the variance explained by each model unique to the model, or did they capture the same information? To evaluate this, we conducted a similar analysis to the one performed before: we predicted the residual choice time with both the cumulative distraction extracted from the network and the MDL. As an illustrative case, we first focused on the best model, Dinov3 small, using the layer that was most predictive of human behavior (its last layer). In this model, the sum of inverse distances between target and distractors had a correlation of 0.54 with human behavior; but when the MDL was added, the correlation increased to 0.71. We then extended the analysis to the 5 best networks and observed that including the symbolic model systematically improved alignment with human data. This suggests that while neural networks do capture an important part of human behavior, they still lack at least some of the explicit, rule-based understanding of geometric relationships that the symbolic model offers. The improved correlation with MDL indicates that symbolic representations contribute unique, structured information not fully captured by the neural network’s learned features alone. Even using the cumulative distraction from each layer of the network and using them all to predict human response time, we get to an R value of 0.73, which improves to 0.81 when adding the MDL. Note that even with this added information, we do not get to the noise ceiling of 0.90, estimated by computing split-half reliability of participants’ residual response time.

When looking at which layers bring about the most human-like representations, we once again see that for most networks (58%), the last layer is the best one (Fig 3E). For 49% of models, the most human-like layer in the first experiment is also the most human-like in this one. Once more, we found that for Vision Transformers trained on image/text pairs, the layer with the most abstract information is situated in the middle layers.

### 2.3 Experiment 3. Can neural networks recognize abstract geometric drawings?

So far, our focus was on the representation of geometrical shapes. However, humans have the capability, from a very early age, to draw and to recognize natural objects depicted using abstract geometric drawings (e.g., a face as a circle with two dots; a man as a stick figure; or a house as a square + triangle) [47–50]. Children’s drawings can be very different from the original item, capturing high-level, abstract features, while ignoring lower-level features, and yet they seem to go to the core of the object they depict: a few geometric shapes suffice for humans to recognize them [4,51–53].

**Fig 4:**
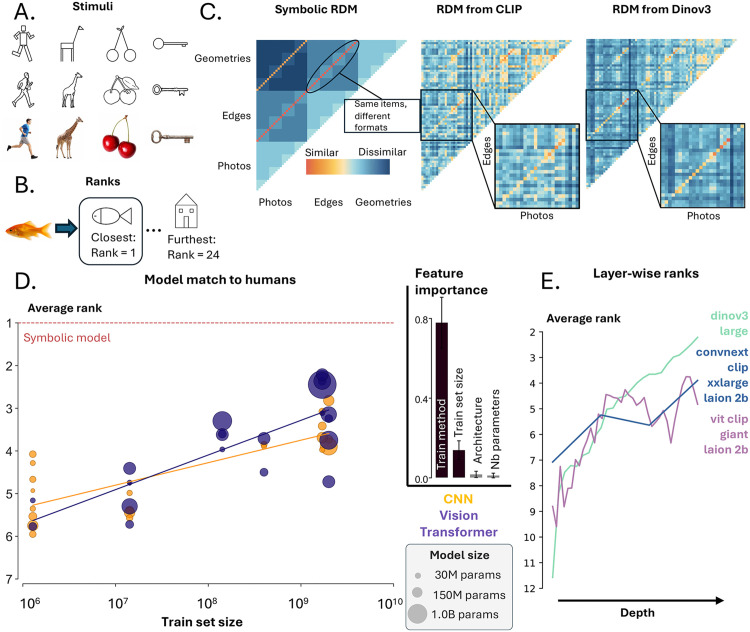
Representation of geometric drawings in different neural networks. **A.** Example stimuli (runner, girafe, cherry, key) across the three renderings (geometric drawings, edges, photos). **B.** Defining the rank: For a given photo, we order the geometric drawings according to their distance to the photo. We then look at the rank of the correct one. In this example the rank is one. **C.** Representation Dissimilarity Matrices (RDMs) for a symbolic model and two neural networks (CLIP and Dinov3). In the symbolic RDM, the small diagonals indicate that same item exhibits a high degree of similarity regardless of format, and the large squares, that photos are more similar to edges than to geometric drawings. Medium squares indicate that items belonging to the same semantic category are also similar to one another. In both neural networks, we see the appearances of small diagonals, suggesting that the networks are able to recognize when the same item is depicted in different formats. **D.** Average rank of the classifiers, plotted as a function of training set size. Color indicates architecture and size of the point the number of parameters of the model. None of the networks achieve the level of the theoretical model. Inset: Feature importance analysis. Contrary to previous experiments, training method appears as the most important predictor of the alignment of a model with human behavior, followed by training set size. **E.** Average rank at each layer of three high-performing networks. Rank is lowest within the deeper layers.

To study this ability, Morfoisse et al. (in preparation) created a set of visual stimuli varying in levels of abstraction. The set comprises representations of 24 items spanning different semantic categories, such as faces, objects or places. These items were represented in 3 different renderings: photos, realistic line drawings of the item’s contours, and minimalistic geometric cartoons made only of segments, triangles, squares and ellipses, for a total of 72 images (see Fig 4A).

To compare human and neural network, we measured how easily neural networks paired photos with geometric drawings. For a given photo of an item, we rank the drawings from closest to farthest to this photo (Fig 4B). We then average the rank of the drawing of the correct item across all items. The smaller that rank is, for every item, the better the network is at representing geometric drawings. We did this for every item, and across all pairs of renderings, testing the generalization from photos to geometric drawings, but also from photos to line drawings and from line drawings to geometric drawings. Because all drawings are immediately recognizable by humans, their rank is always 1, i.e., humans can pair each geometric drawing with the corresponding photo or contour.

To visualize the representations of the images, we computed the Representational Dissimilarity Matrices of the neural networks, and defined a symbolic RDM for illustration purposes (Fig 4C). The symbolic RDM is defined such that it takes into account similarity from different sources. The presence of the sub-diagonals indicates the similarity between the same item in different formats, while the big squares indicate the similarity between different items inside a certain rendering, with photos being farther from minimalist cartoons than line drawings. The medium squares indicate the increased similarity between items belonging to the same semantic category.

Fig 4C shows that both networks, CLIP and Dinov3, appear to demonstrate a capacity to generalize between different renderings. Indeed, the sub-diagonals we observe, although much less pronounced than in the symbolic RDM, show that the same items, in different formats, are close to one another. This is true not only between photos and line drawings, as shown Fig 4C, but also between line drawings and geometric drawings, and, to a lesser extent, between photos and geometric drawings.

In this analysis, as shown in Fig 4D, we observed that none of the tested networks achieved human-level performance on this task. Still, the top-performing network, Dinov3 base, reached an average rank of 2.2, which shows an impressive capability of generalization across formats. As before, a clear overall trend emerged: networks trained on larger datasets generally showed better generalization across renderings.

Similar to what we did in the previous experiments, we used ridge regression to predict a network’s average rank using its training set size, number of parameters, architecture type and training method. Surprisingly, the training set size, while still a significant predictor, was not the most predictive feature (${\Delta R}^{\text{2}}=\text{0}.\text{1}\text{4}\;\pm \text{0}.\text{0}\text{5}$). Instead, the training method appeared as the main contributor (${\Delta R}^{\text{2}}=\text{0}.\text{7}\text{8}\;\pm \text{0}.\text{1}\text{3}$), suggesting that in this case, the dino and CLIP training methods might surpass classification. To determine which training method yields the best generalization, we examined their effects separately rather than jointly: we looked at the importance of the indicator variable for each training method in the regression, not the importance of all three indicator variables together. In this case, we find that the indicator variable for CLIP is not a significant predictor of alignment. By contrast, both classification and dino were significant predictors, but with opposite effects: classification had a positive weight (indicating lower abstract capabilities), whereas dino had a negative weight. Hence the contributions of the varying training methods to a network’s abstract capabilities would be, in order, dino as the best one, followed by contrastive learning (CLIP), and then classification. However, once again, some amount of caution needs to be kept as training method is heavily correlated with the size of training dataset (supervised: R = -0.69, CLIP: R = 0.50, dino: R = 0.25). As in the previous experiments, we complemented this analysis with a linear mixed-effects model including random intercepts for training method and backbone architecture. This model revealed a significant effect of training dataset size (β = −0.16, p = 0.036), indicating that networks trained on larger datasets tend to achieve better ranks (i.e., lower values) in this task. In contrast, the number of parameters did not significantly predict performance (p = 0.59). The random-effects estimates showed non-zero variance associated with training method (σ² = 0.33), suggesting systematic differences between training methods, whereas architecture explained no additional variance. Allowing the effect of dataset size to vary across training methods did not improve model fit (p = 0.29), indicating that the influence of dataset size on performance is broadly consistent across training schemes.

Fig 4E shows that for three networks, the best layer was once again in the last ones, consistent with what we found in the previous experiments. However, across all models, this was not the case as often as with the other experiments, as the last layer was the best one for only 49% of the models tested. Looking at the consistency of the most abstract layer across experiments, we again saw a difference with the other experiments, as the best layer was the same one as in the first experiment only 37% of the time, and 56% of the time for the second experiment.

## 3. Discussion

This paper explores the capacity of deep neural networks (DNNs) to model human behavior across three distinct tasks involving abstract geometric shapes. Our findings indicate that while DNNs can capture some aspects of human geometric perception, they still have limitations in fully replicating human geometric abstraction.

In our first task, focusing on quadrilateral representation, we compared Representational Dissimilarity Matrices (RDMs) from human behavioral data [54] with those from both neural networks and a symbolic model [3]. Interestingly, neural networks offered a better explanation of human data than the symbolic model. When both models were used to predict human behavior, the symbolic model provided no additional predictive power. This suggests that DNNs effectively capture the underlying geometric primitives that humans might use to represent quadrilaterals.

We obtained similar results in our second experiment, where neural networks accurately modeled the difficulty humans experienced in a delayed match-to-sample task with geometric shapes [15], again outperforming the symbolic approach. However, in this case, the symbolic model did add predictive power to the neural network when predicting human behavior, showing that it still captured some information not fully encompassed by the DNNs.

In our final experiment, we tested the networks’ ability to recognize images with varying levels of abstraction: from photos to detailed drawings to minimalistic cartoons drawn using only a few geometric shapes. While the tested models failed to achieve human-level performance, the most effective networks did demonstrate a genuine capacity for generalization across different formats. This result is consistent with prior work showing that DNNs trained on natural images develop a sensitivity to shape that extends to line drawings and silhouettes [55,56]. Specifically, Singer et al. [57] showed that fine-tuning the late layers of a network on ImageNet-Sketch [58] improves correlation with human behavior.

These results connect to a long tradition of using artificial neural networks to model human vision. Artificial neural networks have long been recognized as promising models of the human visual system. For instance, Yamins et al., (2014) showed that the ability of deep convolutional networks trained for object recognition can predict neural responses along the ventral visual stream. Since then, efforts have focused on identifying architectures that best capture the computations of this pathway, as exemplified by benchmarking initiatives such as Brain-Score [59]. Among these, the CORnet family of models [46] represents a targeted attempt to approximate ventral stream dynamics. However, Sablé-Meyer et al. [3] revealed that even the leading models at the time failed to adequately capture human perceptual judgments of geometric shapes. More recent results show that some networks can acquire geometric abilities [26], a finding that our study replicates and extends. This raises a critical question: what drives the emergence of such geometric abilities in artificial models?

To better understand which factors contribute to these abilities, we regressed the correlation of each model with human behavior against various model parameters across all three tasks. In the first two experiments, the main factor driving the quality of alignment to human behavior was the size of the training dataset. In the third one, training method was the most important factor.

The importance of dataset size to achieve human like capabilities is consistent with the broader scaling laws that govern deep learning. Over the past decade, the most significant advances in artificial intelligence have not stemmed from radically new architectures or training paradigms, but primarily from training increasingly large networks on increasingly vast datasets [60–62]. When looking at alignment with neural data, a similar phenomenon appears; bigger networks correlate better with brain activity [63–67]. Because model size and dataset size are often correlated, Gokce & Schrimpf [66] systematically manipulated these factors and found that the increase in alignment was driven primarily by larger training datasets, with model size playing a lesser role. Raugel et al. [67], using a complementary approach, reported that both model size and dataset size can independently contribute to alignment, suggesting that scaling the model itself also provides benefits. Taken together, these results support the view that dataset size is the dominant factor shaping neural alignment, and that model size also plays a role. This conclusion also aligns with prior work showing that training dataset size primarily drives alignment with human behavior in vision tasks [68,69].

Dataset size, however, is likely to also correlate with the richness and diversity of the images seen during training, making it difficult to disentangle the contribution of scale from that of dataset composition. Networks trained on datasets of similar sizes could differ a lot in their alignment to neural data depending on the diversity of the images [70]. It is worth noting that the main effect of visual diet (when disentangled from the size of the training dataset) was reported primarily for relatively small datasets (sizes on the same order of magnitude as ImageNet‑1K), and the models whose performance was most strongly affected by visual diet were trained on highly restricted domains (e.g., models trained exclusively on faces) and therefore operated within a much narrower visual scope.

Additionally, there might be other confounds present that demand caution when interpreting this result. We focused on the impact of four factors: architecture, number of parameters, training objective and size of the training set. These factors have the advantage of being both easily quantifiable, and to have been previously identified as having an impact on the alignment of a neural network with human behavior [31,35,68,69]. Still, we cannot exclude that other differences might have had an impact, such as differences in training schemes or in loss function between networks with the same general training objective (e.g., between Dinov2 and Dinov3).

Could Vision Transformers be better models of human vision than CNNs? While the ViT vs. CNN variable was initially a significant predictor of alignment with human behavior (Figs 2D and 3D), this effect vanished when focusing only on recent architectures, as many older CNNs do not benefit as much from vast amount of data than their newer counterparts. Some studies do suggest that Vision Transformers better match human perceptual biases—favoring shape over texture, for instance [31,35,71]. However, other work finds no consistent advantage in terms of human alignment when controlling for dataset and training objective [69].

Concerning the impact of the training method, our results are not definitive. Training method and dataset size are strongly correlated: models trained on image classification tasks were typically trained on smaller datasets, whereas self-supervised approaches such as DINO or CLIP generally used much larger datasets. In the first two experiments, training method was not a significant feature, but in the last one, it was the most important feature. In all the tasks, models trained on image classification tasks tended to perform worse than those trained with self-supervised approaches such as DINO or CLIP. However, this difference was largely explained by disparities in dataset size rather than by the training method per se, except in the third experiment.

While no network appears to completely outperform all others and stand out as the single best model of human behavior, the results across the different experiments are quite correlated. In our data, when ranking the models according to their correlation with human behavior, the rank-orders are indeed concordant across experiments: the first and second experiments show the strongest agreement (r = 0.67), and the results of the third experiment also correlate with those of the first and second experiments, although to a lesser extent (r = 0.46 and r = 0.60, respectively). Consistently strong performers are the models trained on the largest amounts of data (LVD-1689M and LAION-2B datasets), while older networks trained on the smallest Imagenet-1k dataset consistently appear as the worst models. Across diverse architectures, training objectives, datasets, and scales, these neural networks seem to converge toward a common, human-like encoding of geometric structure. This aligns with the Platonic representation hypothesis [72]: as they gradually get better, different systems tend to encode the same underlying regularities of the world, ultimately converging towards a unified internal representation. One possibility is that this shared representation relies on high-level structural features of shape, such as shape skeletons, which have been proposed to underlie human shape perception [73,74]. However, this account seems unlikely to fully explain our findings: Sablé-Meyer et al., [9] reported that skeletal descriptions provided a poor fit to human behavior on quadrilateral perception, the very task in which we observe the strongest alignment between networks and humans.

Even though the networks accurately modelled human behavior in the first task, they left some variance unexplained in the next two tasks. Specifically, the aggregated group-level pattern of results used as our modeling target was highly reliable, as judged from its split-half noise ceiling, and yet the models did not achieve to fully capture all of this explainable variance. (We note in passing, as a limitation, that we focused solely on group-average results; indeed, the reliability of individual judgments was relatively low, mostly due to a small number of within-subject trials, and prevented us from studying inter-individual variability; see S1 Text for discussion).

This residual, unexplained variance might reflect a fundamental difference between humans and the tested networks: here, we studied models that were solely trained to passively process images. Unlike the models, humans can not only recognize, but also generate shapes. In our first task, the capacity to perceive abstract features such as right angles, parallel lines or symmetry, might be sufficient to account for human behavior. In contrast, the subsequent tasks might require the representation of sequential steps involved in producing a shape. In the second task, the minimum description length (MDL) metric quantifies the number of steps necessary to construct a shape from a limited set of primitives, while in the third task, minimalist cartoons are composed by combining geometric elements such as triangles or rectangles. Both tasks could thus engage the capacity to draw—or at least to mentally simulate drawing—a process not present in the networks we tested. As such, a missing component of those models might be an ability to engage and interact with the visual inputs they are presented, a feature which might be essential to create systems with human-like intelligence [75]. Consistent with this view, adding simple drawing actions such as drawing lines and boxes on images has been shown to substantially improve spatial reasoning in neural networks [76]. A complementary explanation has recently been proposed: current vision–language models appear to lack visually grounded sequential processing, failing to maintain coherent representations across multiple perceptual steps [77].

These shortcomings are consistent with a broader pattern of failures observed, even in the most advanced neural networks, on tasks that are effortless for humans. For instance, recent studies reveal that state-of-the-art models still make systematic errors in visual arithmetic and geometric reasoning [78,79], see illusions where none exist [80], and exhibit striking deficits in spatial intelligence across multimodal systems [81].

Despite these limitations, it is worth considering the possibility that further increases in scale could enhance alignment. It has been suggested that humans’ unique cognitive abilities may emerge from an increase in information capacity [82]. Likewise, networks capable of making use of greater volumes of information, i.e., benefiting from bigger training datasets, could get closer to human-like capacities. In our results, alignment appeared to saturate in the first experiment, but not in the second and third tasks, suggesting that additional data might still yield higher alignment. Indeed, while Gokce & Schrimpf, [66] found that neural alignment reached a plateau with regards to scale, it was not the case for behavioral alignment—supporting the possibility that larger models trained on more data may achieve representations even closer to those observed in humans.

Of course, the amount of visual information processed by these networks during training is orders of magnitude greater than what a human could experience from childhood to adulthood [83]. Therefore, the acquisition of abstract visual concepts by these networks cannot accurately model human development at the scale of human life. However, contrary to artificial neural networks, the infant brain comes wired with considerable biases for efficient visual perception [84,85]. Decades of developmental research have documented such predispositions, ranging from a preference for face-like stimuli [86] to early sensitivities to numerical and spatial regularities [87]. These biases enable infants to extract meaningful structure from very limited data, in sharp contrast with the billions of images some of these models are trained on. From this perspective, the training of the models we test here might not represent individual development, but rather the appearances of biases that were sculpted by natural selection over evolutionary time scales [88,89].

A further concern is whether the apparent alignment between humans and neural networks is due to a confound: the presence of geometric shapes in the training dataset. More data naturally increases the likelihood of including abstract geometric stimuli, graphs or even math problems in the training set. At the scales we examined (up to 2 billion images), it is impossible to manually control for the presence of specific stimuli. Even if there were no explicit abstract shapes in the training set, there are at least man-made objects and structures with right angles and parallel lines. This scaling effect could therefore reflect mere exposure to geometric stimuli, including some that are very close or identical to those used in our tests.

Even if the networks had been trained in part with geometric shapes, the comparison between humans and neural networks would remain meaningful: the participants in the behavioral studies (adults living in Western countries) also had extensive exposure to such images throughout their lives. However, drawing a purely connectionist explanation for human performance on geometric tasks demands caution. While neural networks may extract geometric regularities from these environments, these environments are themselves products of human cognition. Early humans generated such structures without prior exposure; for neural networks to truly account for this ability, they would need to be trained on datasets devoid of such stimuli.

The observed convergence between human and artificial performance invites two contrasting interpretations. A first interpretation emphasizes the explanatory sufficiency of large-scale neural networks. According to this view, the ability of deep networks to master domains previously thought to require a symbolic language renders such theories unnecessary [22], or challenges their usefulness as models of cognition [21].

An alternative interpretation, however, which we favor, holds that symbolic theories remain indispensable as a high-level description of the computations implemented by neural systems [23]. Symbolic frameworks—such as the Language of Thought (LoT) proposed in prior work [1–3,15,90]—should not be understood as competing with neural implementations but as formal descriptions of the underlying computational logic. Neural networks may thus instantiate, in a distributed and implicit manner, the representational structures that symbolic theories make explicit, much like biological brains might do.

Evidence from natural language processing supports this interpretation: language models appear to recover some hierarchical and syntactic regularities consistent with linguistic theory, even in the absence of explicit symbolic rules [42,43,91–93]. Similarly, in the case of geometric perception, the absence of explicit symbolic operations in neural networks does not preclude the emergence of internal representations that functionally resemble a LoT.

As noted by Sablé-Meyer et al. [9], understanding geometrical perception at the neuronal level remains an open challenge. The present study does not claim to resolve this issue but instead proposes a set of computational ingredients that allow a neural network to approximate such processing. We suggest that neural networks may serve—although imperfectly—as models of the underlying neural mechanisms. Analyzing their inner mechanisms may lead to a sharper understanding of how symbol-like manipulations emerge in neural networks and inspire future neuroscience research, as was recently the case for the neural mechanisms of invariant written word recognition [44,94,95].

In sum, our results indicate that, with a massive scale-up of training set size, artificial neural networks come close to approximating the human representation of geometric shapes. While current models remain imperfect proxies of human cognition, they may provide a useful computational framework for probing the mechanisms of abstract visual reasoning. An important challenge for future work will be to improve the interpretability of these models, so that their internal computations can be mapped onto identifiable cognitive operations [96–98]. Progress in this direction will be essential if these systems are to offer genuine insights into the structure of human thought.

## 4. Materials and methods

### Experiment 1

#### Behavioral experiment.

Experiment 1 used data from a previously published study [9]. There were 330 online participants (142 female, 177 male, 11 other; mean age = 51.1 years, SD = 16.8).

On each of 110 trials (11 × 10 ordered pairs of distinct shapes), participants viewed a 3 × 3 grid containing nine quadrilaterals presented in white on a black background. Eight shapes were identical up to rotation and scaling, and one geometrically distinct “intruder” had to be identified by mouse click. Shapes were drawn from a set of 11 quadrilaterals matched for average pairwise vertex distance and bottom-side length. For each trial, scaling (0.85–1.15) and rotation (−25° to 25°, excluding 0°) values were sampled without replacement to prevent perfectly vertical or horizontal alignments. Trial order was randomized with constraints preventing consecutive repetition of the same reference shape and preventing the previous reference from serving as the next intruder. Visual and auditory feedback indicated response accuracy. For more details see [9].

#### Behavioral measure of dissimilarity.

As in Sablé-Meyer et al., [9], the representational dissimilarity between two shapes was estimated as the average success rate divided by the average response time for trials when one of these shapes was target, and the other was the distractor. The more dissimilar two shapes are, the higher the success rate will be, and the shorter the response time, thus yielding a high overall value. Average success rate and average response time were pooled across participants.

To estimate the noise ceiling, we computed the Pearson correlation between the RDMs estimated from 2 disjoint halves of the participants. We repeated this operation 100 times and took the average. See S1 Text for a discussion on noise ceiling estimates.

#### Symbolic model measure of dissimilarity.

Following [9], for the symbolic model, each shape was represented by a vector encoding its discrete geometric properties. The feature space comprised 4 types of properties: right angles (one feature per angle; 4 in total), angle equality (one feature per pair of angles; 6 in total), side-length equality (one feature per pair of sides; 6 in total), and side parallelism (one feature per pair of sides; 6 in total). All features were defined using a tolerance criterion (e.g., angles slightly deviating from 90° were still classified as right angles), with the tolerance fixed at 12.5%, a value previously fitted to independent behavioral data [3].

Pairwise dissimilarity between shapes was computed as the difference in the number of features. Shapes sharing many geometric properties (e.g., a square and a rectangle) were therefore close in this space, as were two highly irregular shapes lacking canonical structure, whereas a regular shape and an irregular one were very dissimilar.

This symbolic model was defined in Sablé-Meyer et al., [3], and proved to be an accurate model of human behavior across multiple experiments, as well as in Sablé-Meyer et al., [9]. The features of interest in the symbolic models were chosen such that these features differ systematically across the 11 quadrilaterals. In principle, it would be possible to define a better instantiation of the symbolic model, for example by according different weights to different features. However, in the present setting, the number of features is large relative to the number of quadrilaterals, which makes it difficult to estimate such weights reliably from the available behavioral data. Allowing feature weights to vary would therefore introduce a strong risk of overfitting, potentially improving fit without capturing more general aspects of human geometric judgments.

For this reason, we use equal weights across features, which provides a simple, parameter-free baseline. This choice avoids adding degrees of freedom, and builds on established models to compare against; developing more flexible versions of the symbolic model would require additional independent data, and is left for future work.

#### Neural networks measure of dissimilarity.

For the neural networks, at a given layer, we computed the Representational Dissimilarity Matrix as follows. For each pair of quadrilaterals (A, B), we extracted the activations evoked by all 36 variants (6 orientations × 6 scales) of shape A and all 36 variants of shape B, computed the distance between each variant of A and each variant of B across all 36 × 36 = 1296 combinations, and averaged these pairwise distances. This mirrors the structure of the human data, in which each participant’s response reflects exposure to multiple trials varying in orientation and scale. This procedure was not applied to the symbolic model, whose features are by construction invariant to scale and rotation. Alignment between model RDMs and behavioral RDMs was quantified using Pearson correlation.

### Experiment 2

#### Behavioral experiment.

Experiment 2 used data from a previously published study [15]. One hundred and twenty-five adults (53 women, 69 men, 3 undisclosed; age range: 20–78 years, median = 44 years) were recruited online via social media and completed the experiment remotely. The shapes used in the experiment were generated by symbolic programs written in a structured drawing language (described below). Each trial comprised an encoding phase, a 2-s blank delay, and a forced-choice phase. During encoding, a target shape was displayed for a self-paced duration: participants held down the space bar for as long as needed to memorize the shape. The duration of the key press was recorded as encoding time. After the delay, participants selected the previously seen shape from a 2 × 3 array of six shapes. The display size differed between encoding and choice phases. Accuracy and response time during the choice phase were recorded. For more details see [15].

#### Behavioral measure of difficulty.

In this case, as representational similarity analysis was not possible, we instead modeled the difficulty of each trial. For humans, the difficulty of a given trial is straightforward: it is the time taken to find the target shape among the distractors (accuracy was not used as it was near ceiling). We then averaged the trials by target shape, giving us a mean trial difficulty per target shape. To estimate the noise ceiling, we computed the correlation between the trial difficulty estimated from two split halves of the participants, repeated this operation a hundred times, and took the average correlation.

#### Symbolic model measure of difficulty.

We modeled symbolic difficulty using the language-of-thought framework introduced by Sablé-Meyer et al., [15], and which was used to generate the shape stimuli. In this model, shapes are generated by compositional symbolic programs written in a structured drawing language.

The language contains (i) drawing primitives that control a pen moving on a plane (e.g., Turn, Move, Trace), (ii) control structures that enable compositional structure (e.g., Concatenate, Repeat, Subprogram), and (iii) a minimal numerical system allowing integer and rational parameters. Programs are represented as syntactic trees composed of these primitives and operations.

Because any given shape can in principle be generated by infinitely many programs, the model additionally assumes that humans favor the shortest program that reproduces the shape. The Minimum Description Length (MDL) of a shape is therefore defined as the length of its shortest generating program in this language. Program length corresponds to the number of primitives (i.e., nodes in the syntax tree).

Following this framework, the hypothesis is that the complexity of a mental representation is the MDL of the target shape, and will impact the difficulty of a trial. For each stimulus, we computed the length of its minimal program and used this value as the symbolic measure of complexity. This measure was shown to be a good predictor of human behavior, above what could be explained by lower level perceptual information [15].

#### Neural networks measure of difficulty.

For neural networks, we estimated the difficulty of each trial based on the similarity between the internal representations of the target and the five distractors. We initially tried several metrics. A first approximation, used by Campbell et al. [26], is to relate response time to the distance between the embedding of the target shape and the centroid (i.e., average embedding) of all the distractors. However, this approach presents a potential limitation. The centroid is strongly influenced by distractors that are far from the target: a single highly dissimilar distractor can shift the centroid away from the target, thereby increasing the target–centroid distance. Yet such a distractor should contribute very little to task difficulty. A highly dissimilar distractor can be rapidly rejected and effectively ignored during visual search, and therefore should have little impact on response time. In contrast, a distractor that is very close to the target should substantially increase difficulty and lead to longer response times. Because the centroid aggregates all distractors equally, it overweights distant distractors and underrepresents the impact of highly similar ones.

We then opted for a second approach using the inverse distance between the target embedding and that of the most similar distractor, under the assumption that similarity to that distractor is the main determinant of task difficulty. While this metric captured cases in which a single distractor was highly similar to the target, it still did not account for the cumulative effect of multiple similar distractors.

We therefore defined a third, more refined measure, cumulative distraction, based on the full set of target–distractor distances. Given the embeddings for a target shape T, and 5 distractors D_1_, …, D_5_, the cumulative distraction is defined as:


$$\begin{array}{c} Distraction=\;\sum_{i=\text{1}}^{\text{5}}\frac{\text{1}}{distance(T,\;D_{i})} \end{array}$$

In this equation, distractors that are a large distance from the target will contribute very little to the cumulative distraction: $\frac{\text{1}}{distance(T,\;D_{i})}\sim \text{0}$, whereas distractors that are very close to the target shape will contribute greatly to the distraction. Additionally, multiple similar distractors can jointly contribute to the distraction.

### Experiment 3

#### Stimuli.

We created a dataset of 72 images, depicting 24 different entities, spanning six semantic categories (faces, bodies, animals, flora, objects, and places), and rendering in 3 different formats (color photos, line drawings, and geometrical compositions). Photos were obtained from freely available images. Line drawings were created by extracting the edges of new photos using the Holistically-Nested Edge Detection algorithm [99], and then manually simplifying the contours to have them look like human drawings. Geometric compositions were created by combining four geometrical shapes—lines, ellipses, triangles and rectangles—using only scaling and rotations, but no other distortions. For each composition, a maximum of ten minimal shapes were used. To validate recognizability, Morfoisse et al. (in prep) conducted a behavioral experiment with 10 independent adult participants using a larger set of minimalist cartoons than those presented here. They only selected images that could be accurately named, with a recognition accuracy above 90%. They ended up with a set of 72 images: 24 photos, 24 line drawings (or edges), and 24 geometric drawings (or minimalist cartoons). (see Fig 4A)

#### Neural networks generalization.

To measure the capacity of a neural network to generalize between formats, we looked at the distances between embeddings of the same items, across different formats. For a given photo of an item, we rank the drawings from closest for farthest (Fig 4B). We then average the rank of the correct drawing across items. We not only looked at the generalization from photo to drawings, but from photos to minimalist cartoons, and from drawings to minimalistic cartoons.

For humans, as all drawings are immediately recognizable, their rank is always 1.

In this experiment, no symbolic dissimilarity measure was derived in the same sense as in Experiments 1 and 2. Instead, the symbolic RDM was defined based on a direct one-to-one correspondence between items across formats, reflecting a priori knowledge about item identity rather than any computed geometric or structural features.

### Neural networks models

#### Model architecture and training.

The networks used in this paper belong to two main architecture categories: the Convolutional Neural Network (CNN) and the Vision Transformer (ViT).

Within the CNN family, we included several modern convolutional architectures. These comprised multiple variants of ResNet [100], ConvNeXt [41], as well as CORnet [46], a recurrent convolutional architecture designed to approximate the hierarchical organization of the primate ventral visual stream.

The full list of neural networks is available in S1 Table.

These networks were trained using different learning paradigms. Some models were trained with supervised learning, using category labels to optimize image classification performance. Others were trained with self-supervised learning, in particular with DINO [101], which learn visual representations without manual category labels. A third group of models was trained with contrastive language-image learning, or CLIP [38], where aligned image-text pairs are used to learn joint visual and linguistic representations.

The networks could be trained on one of six datasets: ImageNet-1k (1.2 million images), ImageNet-21k (23 million images), LVD-142M (142 million images), OpenCLIP (400 million image-text pairs), LVD-1697M (1.7 billion images), and LAION-2B (2 billion image-text pairs).

#### Implementation.

All neural networks were downloaded from two python libraries, Transformers [102] and timm [103], except Cornet which was downloaded directly from the dedicated GitHub repository [18]. All models were run in the PyTorch framework.

Input images were resized and normalized according to the preprocessing conventions associated with each pretrained model. In practice, this involved resizing images to the resolution expected by the model (typically 224 × 224 pixels) and normalizing pixel values using channel-wise means and standard deviations defined during training.

All networks were evaluated in inference mode with pretrained weights, on GPU. Feature representations were extracted from multiple layers along the hierarchy of each model. For convolutional neural networks (e.g., ResNet, ConvNeXt, and CORnet), activations were extracted after each successive convolutional stage of the network. For transformer-based architectures, representations were extracted after the multilayer perceptron (MLP) block of each transformer layer. We used Euclidean distance to measure distances between embeddings. Equivalent results were obtained with cosine distance and correlation (S1 Fig), which are less sensitive to dimensionality.

### Feature importance analysis

To quantify which properties of neural networks best explained their correspondence with the target measure, we performed a feature importance analysis based on linear modeling and permutation tests.

#### Ridge regression on correlation.

We modeled the relationship between model characteristics and their behavioral alignment score using a ridge regression, fit separately for each experiment. The dependent variable was the Fisher-transformed Pearson correlation in Experiments 1 and 2, and the average rank in Experiment 3. The predictors included the number of model parameters, the training dataset size, the network backbone architecture, and the training method. Categorical variables (backbone and training method) were one-hot encoded. Continuous predictors (number of parameters and training dataset size) were standardized before fitting the model. Ridge regularization was used to stabilize coefficient estimates in the presence of correlated predictors.

#### Permutation-based feature importance.

Feature importance was estimated using a grouped permutation procedure. For each predictor, all columns associated with that feature (e.g., all dummy variables corresponding to a categorical factor) were jointly permuted across observations while keeping the remaining predictors unchanged. The ridge model was then used to recompute the coefficient of determination (R²) on the held-out fold. The importance of a feature was defined as the decrease in R² relative to the baseline obtained with unpermuted data. This procedure was repeated 30 times per fold, yielding a distribution of performance drops for each feature, averaged across folds and permutation repeats.

For Experiments 1 and 2, model fitting and importance estimation were performed using 5-fold cross-validation over participants: the ridge regression was fit on correlations derived from 80% of participants, and permutation importance was evaluated on the held-out 20%. A feature was deemed significant if its mean performance drop exceeded a Bonferroni-corrected z-threshold in all five folds. For Experiment 3, cross-validation was not applied as there are no participants over whom to define folds; significance was instead assessed within the single fit using the same Bonferroni-corrected z-threshold.

#### Linear mixed-effects models.

To further quantify the contribution of dataset size and model scale while accounting for architectural and training differences, we fitted linear mixed-effects models using the lme4 library [104]. The dependent variable was again the correlation score. Fixed effects included the logarithm of the training dataset size and the logarithm of the number of model parameters: correlation ~ log(training set size) + log(number of parameters) + (1|architecture) + (1|train method).

Random intercepts were included for both training method and backbone architecture, allowing the baseline correlation to vary across these factors. We also tested a model allowing the effect of dataset size to vary across training methods by including a random slope for dataset size. Model comparisons were performed using likelihood-ratio tests. We report the fixed-effect coefficient (β) and its associated p-value for each predictor.

## Supporting information

S1 Fig Same as Fig 2D, but using A: cosine similarity, B: correlation. (TIFF)

S2 Fig Spearman correlation of the model RDMs with human behavior as a function of the Pearson correlation of the model RDMs with human behavior. (TIFF)

S3 Fig Same as Fig 3D, controlling for more confounds: spatial frequency, number of extremities, intersection where two lines meet, intersections where three lines meet, intersections where four or more lines meet, disconnected parts, and presence of closed shape or not. (TIFF)

S1 Table List of the models used, with their main characteristics: number of parameters, train set, architecture and training method. ViT: Vision Transformer; CNN: Convolutional Neural; Network; CLIP: Contrastive Language-Image Pre-training; in1k: Imagenet-1k; in22k: Imagenet-22k; Laion-2b: Large-scale Artificial Intelligence Open Network
(TIFF)

S2 Table List of the models used, and the layer that provided the best alignment with human behavior in each experiment. (TIFF)

S1 Text Discussion on the noise ceilings. (PDF)
