# Supplementary material for: Can neural networks model the human perception of geometric shapes?
Source: PLoS Comput Biol. 2026 Jul 27;22(7):e1014554. doi: 10.1371/journal.pcbi.1014554 (PMC13440860; doi:10.1371/journal.pcbi.1014554)
Supplement: Supplementary file 1 [file pcbi.1014554.s006.pdf]

S1 Text: noise ceiling estimation

In the main text, noise ceilings were estimated by a split-half procedure: participants were randomly partitioned into two disjoint halves, a Representational Dissimilarity Matrix (RDM) was computed from each half, and the Pearson correlation between the two resulting RDMs was averaged over 100 random splits. This yielded noise ceilings of r = 0.99 in Experiment 1 and r = 0.90 in Experiment 2.

An alternative is a leave-one-subject-out (LOSO) estimator, in which each participant's RDM is correlated with the group RDM computed from the remaining n–1 participants, and the resulting correlations are averaged. Applied to our data, this yields noise ceilings of r = 0.77 (Experiment 1) and r = 0.41 (Experiment 2).

The two estimators target different quantities. The quantity of interest in our analyses is the group-level representational geometry, that is, the dissimilarity structure that emerges once behavioral measurements have been aggregated across participants. Single-participant RDMs are dominated by measurement noise, since each pairwise dissimilarity is derived from a small number of trials. The split-half procedure quantifies the reproducibility of the aggregated RDM that actually serves as the target of the model comparison, whereas the LOSO procedure conflates the measurement noise of individual RDMs with the reliability of the group representation, and is therefore largely driven by the former. Because our neural networks comparisons operate at the group level, the split-half estimate is the appropriate reference.
