# Supplementary material for: Can neural networks model the human perception of geometric shapes?
Source: PLoS Comput Biol. 2026 Jul 27;22(7):e1014554. doi: 10.1371/journal.pcbi.1014554 (PMC13440860; doi:10.1371/journal.pcbi.1014554)
Supplement: Supplementary file 2 [file pcbi.1014554.s008.pdf]

The authors have made substantial improvements to the original manuscript, particularly through the addition of a dedicated Methods section and a clearer explanation of symbolic models in the Introduction. The Discussion has also been significantly strengthened, and the inclusion of additional analyses has improved the clarity of the results.

The manuscript makes a clear and valuable contribution in demonstrating that modern artificial neural networks exhibit increasingly human-like geometric shape representations. This is a self-contained and interesting result that will be of relevance to the journal's readership.

However, despite these improvements, the manuscript remains insufficiently motivated, and the overall narrative is still difficult to follow. In addition, several methodological concerns persist that should be addressed before the manuscript can be considered suitable for publication.

Color code:

- Reviewer's comments round 1
- Authors' reply round 1
- Reviewer's comments round 2

1. Major comments:

(1.1) The Introduction suggests that the authors aim to compare the alignment of symbolic and deep learning–based AI models with human geometric shape perception. However, the manuscript does not provide a clear definition of what is meant by "symbolic," nor does it explain how symbolic models are conceptually related to the language-of-thought framework and how this differs from deep learning–based approaches.

Thank you for the remark. We added this definition in the introduction: "By symbolic, we refer to representational systems composed of discrete elements that can be composed into complex expressions according to a set of explicit rules, a grammar." (page 3 lines 27-29).

The authors now provide a precise definition of a symbolic model in the Introduction. While technically accurate, the definition remains quite abstract and may be difficult for a broader readership to grasp. Since the manuscript draws comparisons with connectionist approaches, it would be helpful to include a parallel definition of a connectionist model that mirrors the structure of the symbolic model definition.

For example, a connectionist model could be described as *a system that learns distributed representations by optimizing continuous connection weights,*

allowing complex patterns to emerge without explicitly specified rules, beyond the implicit constraints imposed by the architecture and training setup. Presenting both definitions in a comparable format would help clarify the key distinctions between symbolic and connectionist models and would also provide an implicit contrast (i.e., a definition by negation) of symbolic models.

Convolutional neural networks (CNNs) and vision transformers (ViT) are known to learn high-level, human-relevant structure (e.g., concept detectors, objectness, or segmentation-like signals), raising the question of how such learned representations differ from what the authors term "symbolic." Indeed, one could argue that certain neural networks trained on large-scale datasets may develop proto-symbolic representations, which complicates a strict dichotomy between symbolic and neural models. For example, Thompson et al. (2024) showed that a relatively simple dual-stream recurrent neural network can encode the abstract relational structure between objects and generalize zero-shot to novel object types–both hallmarks of symbolic cognition.

If both approaches are capable of encoding high-level abstract information, the basis on which they are being distinguished in the present study requires further clarification. If the primary distinction is simply that symbolic models rely on hand-coded representations whereas neural networks learn distributed representations through training, then the conceptual relevance of comparing symbolic and deep-learning models becomes less clear. Clarifying these distinctions and explicitly situating both modeling approaches within their respective theoretical frameworks would strengthen the conceptual grounding of the paper and help readers better understand the motivation for the comparisons made between ANNs and symbolic models.

Neural networks learn distributed representations; by design, they do not make use of explicit hand-coded symbols like the symbolic model. This, however, does not preclude the emergence of what the referee would call proto-symbolic representations. This is what we discuss p.21 lines 12-16: "Symbolic frameworks […] should not be understood as competing with neural implementations but as formal descriptions of the underlying computational logic. Neural networks may thus instantiate, in a distributed and implicit manner, the representational structures that symbolic theories make explicit, much like biological brains might do."

We believe that the result from Thompson et al. (2024), cited by the reviewer, reinforces our argument: a neural network can account for behaviors previously thought to require symbolic machinery — in their case, counting; in ours, geometry. The conceptual value of comparing both modeling approaches lies precisely in asking whether neural networks can capture the same abstract structure that symbolic models make explicit, and to what degree. We have expanded the introduction (p. 3, lines 38–42) to better situate this debate.

The authors have satisfactorily addressed this point; however, the Introduction would still benefit from further refinement (see major comments 1.8).

(1.2) It is also important to note that the symbolic models employed across the three experiments differ substantially in their assumptions and implementations. As a result, while it is informative to assess whether ANNs outperform a given symbolic model within a specific experiment, comparisons across experiments do not support general conclusions about whether symbolic models or ANNs better approximate human geometric shape judgments. In principle, a different or more nuanced instantiation of a symbolic model could capture human judgments equally well or even outperform ANNs. This raises questions about whether the specific symbolic models employed here provide a meaningful benchmark for assessing alignment with human geometric perception. Moreover, the manuscript offers limited discussion of the symbolic models' results and their comparison with ANNs. The authors may therefore wish to reconsider whether including symbolic models as a baseline is informative in this context, or alternatively, to more clearly articulate the intended role and interpretive value of symbolic models within the study as suggested in the previous comment.

We agree that the symbolic models vary across experiments and that comparisons across experiments do not support general claims about symbolic vs. neural approaches. We also agree that a more nuanced symbolic model could, in principle, perform better.

That said, the symbolic models used here do provide a meaningful baseline: they have been shown in prior work to capture human behavior well (Sablé-Meyer et al., 2021, 2022, 2025). We have added more information in the Methods section to explain better what are the symbolic models, and why they present a good, although imperfect, model of human behavior (p. 22, line 43 to p. 23 line 6, p. 23 line 49 to p. 24 line 16).

The key finding here is not that neural networks outperform all conceivable symbolic models, but that they outperform models which were previously considered good accounts of human behavior. This is informative precisely because it suggests that modern networks may help bridge the gap between connectionist and symbolic theories of geometric shape perception, something older models did not achieve. We have added a paragraph in the introduction (p. 4, lines 18–24) to make this interpretive framing explicit.

I appreciate the authors' clarification and the expanded discussion. However, I remain unconvinced by the claim that the present findings "bridge the gap" between connectionist and symbolic accounts, particularly given the limited understanding of the mechanisms underlying human geometric shape perception. Demonstrating that modern ANNs better approximate human behavior than prior symbolic models does

not necessarily imply that these ANNs implement more “symbolic-like” representations or that both approaches converge onto similar representational structures, as mentioned in the Discussion.

Relatedly, the connection between the symbolic models used in this study and the language-of-thought framework remains unclear. While this link is suggested in the Introduction, it is not explicitly specified. It would be helpful for the authors to clarify whether these symbolic models are theoretically grounded in the language-of-thought hypothesis or instead constitute ad hoc, rule-based formulations. If the latter, their contribution requires further clarification. In particular, since the current symbolic models were human-designed, it is perhaps unsurprising that they align with human behavior better than old CNN models.

While the manuscript convincingly shows that modern ANNs provide increasingly human-like representations, the added value of including symbolic models in the current manuscript to advance our understanding of human shape perception or ANN representations is currently unclear.

(1.3) One of the main findings of the manuscript is that the size of the training dataset primarily predicts the alignment between human behavior and computer vision models. However, training set size is confounded with several other factors that could likely enhance the correspondence between humans and ANNs (see Supplementary Table 1). Larger datasets are typically associated with different training objectives (e.g., supervised ImageNet vs. contrastive CLIP or self-supervised DINO/DINOv3), substantial differences in dataset identity and visual richness (e.g., ImageNet-1k vs. ImageNet-22k or large-scale web/video corpora), increases in model capacity, and variations in pretraining pipelines, optimizers, and preprocessing. As a result, the apparent advantage of larger training sets may reflect a combination of these factors rather than dataset size per se. This confound represents a substantial limitation of the study and complicates the interpretation that training set size is the primary factor of human–model alignment in geometrical shape perception. Although this issue does not admit an easy solution given the available model zoo, it calls for a more cautious interpretation of the results, stronger methodological controls (see next comment), and a more explicit discussion of the relevant confounding factors.

It is true that there are confounds present between the various characteristics of the different neural networks, and, as the reviewer noted, there are some confounds that can hardly be controlled for. We have implemented the mixed effect model suggested in the next comment, and spent more time in the discussion to properly acknowledge, and when possible address, these limitations (p. 18, lines 31-38)

I appreciate the efforts of the authors in implementing the suggested mixed-effects model and in improving the Discussion to more clearly acknowledge these limitations.

(1.4) Relatedly, it would be important to clarify whether the ridge regression analysis was cross-validated. If the regression was fit on the full dataset without cross-validation, the results are better interpreted as descriptive rather than predictive, and reporting standardized coefficients and uncertainty estimates may be more informative than permutation importance alone. However, I recommend using cross-validated feature-importance estimates (e.g., permutation importance on held-out folds) to reduce optimistic bias. Depending on how alignment is computed, this could be implemented via leave-one-participant-out cross-validation, or if alignment is based on an aggregate human RDM, using bootstrap/cross-validation over stimuli (e.g., split-half or k-fold over images/conditions to build human and model RDMs).

We thank the reviewer for this suggestion. For Experiments 1 and 2, we implemented cross-validation over participants as suggested; Experiment 3 has no participants over whom to fold. Cross-validation over stimuli was considered but not feasible: Experiment 1 has only 11 shapes, making held-out subsets too small for stable estimates, and in Experiment 3, the rank-based measure is computed relative to the full stimulus set and cannot be evaluated on a subset.

The authors have largely addressed this point.

I suggest complementing the current ridge regression analysis with a hierarchical linear mixed-effects model in which the dependent variable is each model's human–model RDM alignment (e.g., the Spearman correlation between the upper triangles of the RDMs). While this approach will not eliminate collinearity among predictors, it provides a useful robustness check to assess whether training set size remains the dominant predictor within clusters of related models and training approaches, and it quantifies how much variance is captured by group-level effects versus dataset size. In particular, mixed-effects modeling can explicitly account for clustered dependence by including random intercepts for grouping variables such as training method and architecture family (e.g., (1|training method), (1|architecture), or potentially (1 + log(training set size) | training method) and (1 + log(training set size) | architecture)). This reduces the risk that systematic differences shared by models trained under the same pipeline or stimulus set are inadvertently attributed to dataset size, as the model can allocate variance to the appropriate group-level terms rather than forcing it into the fixed effects.

Thank you for the recommendation. We added the mixed-model analysis (see Results, p. 7 line 43 to p.8 line 5, p.11 lines 31-37, p. 15 lines 16-25), and train set

size was still a significant predictor of alignment, controlling for these group-level effects.

This point has been satisfactorily addressed through the inclusion of the proposed analysis.

(1.5) Training set size is closely correlated with the richness and diversity of the visual diet, as larger datasets typically contain more varied and semantically rich stimuli. Importantly, Conwell et al. (2024) show through controlled analyses that it is this representational richness, rather than dataset size per se, that primarily drives increased alignment between human and model representations. This constitutes a confound that can hardly be resolved using statistical tricks in the current study, and it should therefore be explicitly acknowledged and discussed by the authors.

This is a good point: as things stand, we cannot control for the distribution of images in the training set. We thank the referee for the Conwell et al reference, which is very relevant; we added it to the discussion (p.18 lines 21-29).

(1.6) A particularly interesting finding of the study is that, in Experiment 3, the authors show that ANNs exhibit sensitivity to pairings between photographs and geometric drawings. However, this result is not entirely novel, and the manuscript would benefit from citing prior related work. For example, Singer et al. (2022) demonstrated that generalization to abstracted images, such as drawings, can emerge in convolutional neural networks trained on natural images.

Indeed, it is an important reference that we missed. Thank you for pointing it out.

(1.7) I note that the noise ceiling estimates in Experiment 1 are very high ($r = 0.99$). The noise ceiling was computed by pooling behavioral measurements across participants within each split iteratively. This means that noise was effectively cancelled across 165 participants within each split. If we assume that each participant can be treated as a distinct model, this procedure effectively removes inter-model (i.e., between-subjects) noise. For ANNs, however, measurements were averaged within each model across different presentations of the same shape, which primarily removes intra-model noise (i.e., within-subject). Thus, although averaging was applied in both cases, the type of noise being cancelled differs between humans and models. This asymmetry may result in an artificially high noise ceiling, effectively setting an overly optimistic benchmark for ANN performance.

This is a critical point that may also affect Experiment 2 and should be addressed more explicitly. One possible approach would be to estimate the noise ceiling using a leave-one-subject-out procedure. On each iteration, a single participant is held out, and an RDM is computed from the remaining (n–1)

participants. A second RDM is computed for the held-out participant, and the noise ceiling is estimated as the average correlation between each participant and the corresponding group-level RDM. This would provide a more comparable estimate to the correlations obtained between ANN models and the group-level human RDM.

(1.8) While the authors' efforts to improve the Introduction and Discussion have strengthened the manuscript, the overall presentation still lacks clarity. In particular, the Introduction remains somewhat difficult to follow and, at times, potentially misleading, primarily due to the absence of a clear and coherent narrative guiding the reader through the paper's objectives.

The manuscript begins by introducing geometric shape perception and then motivates the idea that human shape perception may be symbolic, drawing on the language-of-thought framework and situating the work within the renewed debate between connectionist and symbolic models. At this stage, the reader may reasonably infer that the primary goal of the manuscript is to arbitrate between these two accounts. However, the subsequent paragraph shifts focus to identifying which ANN hyperparameters yield greater human alignment at the behavioral level. The manuscript then returns to symbolic models as a baseline, framing the goal as evaluating whether contemporary neural networks can match or exceed symbolic models previously shown to capture human behavior. This is followed by the introduction of CNNs and ViTs, further broadening the scope.

Overall, the progression of ideas lacks a clear through-line, and the specific research question and contribution of the manuscript remain somewhat ambiguous. I believe the manuscript would benefit from substantial revision to more clearly define its goals, better situate the theoretical context, and improve the organization of both the Introduction and Discussion. Strengthening the narrative structure would make the paper significantly easier to follow and interpret.

(1.9) I believe the Discussion would benefit from considering the possibility that the alignment between modern ANNs and humans is driven by high-level visual features. This interpretation is consistent with prior work suggesting that shape perception may rely on abstract structural properties of objects (e.g., shape skeletons; Chaz Firestone & Brian Scholl, 2014; Valeria Ayzenberg & Stella Lourenco, 2022).

(1.10) I encourage the authors to include a dedicated Data Availability section within the Methods. This section should explicitly state whether the data are openly available and, if so, provide hyperlinks and clear information on how they can be accessed. Doing so would align the manuscript with the open science principles of PLOS Computational Biology and enhance transparency and reproducibility.

2. Minor points and clarity:

Comments (2.1) to (2.5), (2.8) and (2.9) are about methods, as well as clarity of the text and the figures. We have adressed these issues by adding a Methods section, and followed the reviewers recommendations on clarity in both text and figures.

(2.1) Page 2 - Paragraph 4: "*We used Representational Similarity Analysis (RSA) to compare the internal representations of humans and neural networks on quadrilaterals*". This is potentially misleading, as it suggests that the human RDMs are derived from neural data. To avoid confusion, it would be clearer to specify from the outset that these are *behaviorally estimated* internal representations of humans (e.g., "behaviorally estimated human representations").

(2.2) Page 3 - Paragraph 2: The authors state that the RDM was derived from participants' "success rates" and reaction times. This terminology may be ambiguous, as cognitive scientists typically interpret "success rate" as accuracy, whereas in the AI literature it can refer to different metrics (e.g., true positive rate). To avoid confusion, it would be helpful for the authors to explicitly define what is meant by "success rate" in this context (e.g., accuracy).

(2.3) Page 3 - Paragraph 2: Related to the point above, it is unclear how the participant RDMs were estimated. Were individual participant RDMs correlated with the model RDMs, or did the authors compute a single RDM aggregated or averaged across participants? Please clarify this in the text.

(2.4) Page 4 - Paragraph 1: It is explained that to compute the ANN RDM the layer with the highest correlation was used, and that for different networks different layers were selected. To improve clarity I suggest adding a table to the supplementary materials with the specific layer used for every model.

(2.5) Page 4 - Paragraph 2: The symbolic model used in Experiment 1 is introduced as representing quadrilaterals based on their inherent geometric properties. While the model is referenced to prior work (Sablé-Meyer et al., 2021), it would be helpful to clearly enumerate all the geometric properties that were considered and how they were encoded. This clarification would aid readers in understanding how distances between stimuli were computed.

(2.6) Page 8 - Paragraph 1: The authors introduce the cumulative distraction method as an alternative to Campbell's approach (2024), which was noted to be sensitive to outlier distractors. However, it is unclear how the cumulative distraction method addresses this limitation. In particular, extreme distractors—those with very large or very small distances—would still contribute equally when distances are averaged, potentially reintroducing the same sensitivity to outliers. Please clarify how this method mitigates the influence of outlier distractors, or otherwise justify its advantages over Campbell's original approach.

We now explain this more thoroughly in the methods section, page 24: “The centroid is strongly influenced by distractors that are far from the target: a single highly dissimilar distractor can shift the centroid away from the target, thereby increasing the target–centroid distance. Yet such a distractor should contribute very little to task difficulty. A highly dissimilar distractor can be rapidly rejected and effeclvely ignored during visual search, and therefore should have little impact on recognition. In contrast, a distractor that is very close to the target should substantially increase difficulty and lead to longer reaction times. Because the centroid aggregates all distractors symmetrically, it overweights distant distractors and underrepresents the impact of highly similar ones.”

This point has been satisfactorily resolved.

(2.7) The authors note a potential confound between luminance and image complexity and attempt to address this by regressing out target luminance from choice time. However, another plausible confounding factor is spatial frequency: more complex images typically contain higher spatial frequency content. The authors may therefore also wish to consider regressing out spatial frequency–related measures, but results should not be affected as spatial frequency will likely be highly correlated with luminance.

This is true. We added an analysis (figure S4) controlling for more confounding factors, including spatial frequency. As the referee correctly predicted, conclusions were unchanged.

I appreciate the inclusion of control analyses for spatial frequency information.

(2.8) Figure 2, 3, and 4: I suggest using more dissimilar colors for panel E.

(2.9) Please clarify what the error bars represent in Figure 3C. In addition, what do the square markers in panel D denote? I assume they correspond to a combination of ANN and symbolic models; if so, this should be explicitly stated and included in the figure legend.

(2.10) Page 3, line 38: The statement that recent advances in AI have rekindled the debate between classical and connectionist theories would benefit from the inclusion of recent references explicitly engaging with this debate. This would help situate the current work within the contemporary literature.

(2.11) Figure 1B: The graphical representation of the symbolic model is somewhat confusing. While it appears intended to depict a syntactic tree structure, representing it as a set of interconnected nodes resembles a connectionist architecture and may blur the intended distinction. Consider revising the figure.

(2.12) Page 5: While the summary of results in the final two paragraphs of the Introduction is useful, it is somewhat lengthy and repetitive. This section could be made more concise and focused.

(2.13) Page 6, line 34: The term “geometric regularity” would benefit from a brief clarification (e.g., whether it refers to equal side lengths, equal angles, or other structural properties).

(2.14) Page 7: The claim that dataset size is the dominant predictor of behavioral alignment is not supported with sufficient statistical detail. Please report relevant statistics (e.g., effect sizes, confidence intervals) to convey the magnitude of the effects. Additionally, clarify whether the permuted feature importance distributions were directly compared across variables.

(2.15) Page 9, line 9: Minor grammatical issue: “the results ‘*remain*’ unchanged.”

(2.16) Page 12, line 15: The specific distance metric should be explicitly stated in the Methods section. If Euclidean distance was used as mentioned in the Results section, it would be helpful to note that it can be influenced by the dimensionality of the feature space, with higher dimensionality typically leading to larger distances. While this may not critically affect the construction of RDMs, acknowledging this point—and ideally confirming that similar results are obtained with alternative distance measures less sensitive to dimensionality (i.e., cosine distance)—would strengthen the analysis. Additionally, please specify how RDMs were compared (e.g., Spearman correlation) to improve clarity and reproducibility.

(2.17) Page 27: A reference to the package used for the linear mixed-effects models appears to be missing and should be included for reproducibility.

References:

- Ayzenberg, V. and Lourenco, S. (2022) Perc eption of an object’s global shape is best described by a mode l of skeletal structure in human infants. eLife 11, e74943
- Conwell, C., Prince, J. S., Kay, K. N., Alvarez, G. A., & Konkle, T. (2024). A large-scale examination of inductive biases shaping high-level visual representation in brains and machines. *Nature communications*, *15*(1), 9383
- Firestone, C. and Scholl, B.J. (2014) “Plea se tap the shape, anywhere you like”: shape skeletons in human vision revealed by an exceedingly simple measure. Psychol. Sci. 25, 377–386
- Singer, J. J., Seeliger, K., Kietzmann, T. C., & Hebart, M. N. (2022). From photos to sketches-how humans and deep neural networks process objects across different levels of visual abstraction. *Journal of vision*, *22*(2), 4-4.

- Thompson, J. A., Sheahan, H., Dumbalska, T., Sandbrink, J. D., Piazza, M., & Summerfield, C. (2024). Zero-shot counting with a dual-stream neural network model. *Neuron*, *112*(24), 4147-4158.

Criteria for publication

In addition to being ethically conducted and scientifically valid, research published in *PLOS Computational Biology* should fulfill each of the following criteria:

1. Originality
2. Innovation
3. High importance to researchers in the field
4. Significant biological and/or methodological insight
5. Rigorous methodology
6. Substantial evidence for its conclusions

Writing the review

The purpose of the review is to provide the editors with an expert opinion regarding the validity and quality of the manuscript under consideration. The review should also supply authors with explicit feedback on how to improve their papers so that they will be acceptable for publication in *PLOS Computational Biology*. As you write, consider the following points:

- What are the main claims of the paper and how significant are they for the discipline?
- Are these claims novel? If not, which published articles weaken the claims of originality of this one?
- Are the claims properly placed in the context of the previous literature? Have the authors treated the literature fairly?
- Do the data and analyses fully support the claims? If not, what other evidence is required?
- Would additional work improve the paper? How much better would the paper be if this work were performed and how difficult would it be to do this work?
- *PLOS Computational Biology* encourages authors to publish detailed protocols and algorithms as supporting information online. Do any particular methods used in the manuscript warrant such treatment? If a protocol is already provided, for example for a randomized controlled trial, are there any important deviations from it? If so, have the authors explained adequately why the deviations occurred?
- Are original data deposited in appropriate repositories and accession/version numbers provided for genes, proteins, mutants, diseases, etc.?
- Has the author-generated code that underpins the findings been made publicly available?
- Does the study conform to any relevant guidelines such as CONSORT, MIAME, QUORUM, STROBE, and the Fort Lauderdale agreement?
- Are details of the methodology sufficient to allow the experiments to be reproduced?
- Is any software created by the authors freely available?
- Is the manuscript well organized and written clearly enough to be accessible to non-specialists?

- Does the paper use standardized scientific nomenclature and abbreviations? If not, are these explained at the first usage?
