# Supplementary material for: Can neural networks model the human perception of geometric shapes?
Source: PLoS Comput Biol. 2026 Jul 27;22(7):e1014554. doi: 10.1371/journal.pcbi.1014554 (PMC13440860; doi:10.1371/journal.pcbi.1014554)
Supplement: Supplementary file 3 [file pcbi.1014554.s009.pdf]

General remarks:

We thank the reviewers for their careful reading of the manuscript and for their constructive comments. Their suggestions helped us improve both the clarity and the methodological description of the work. In particular, we have added a dedicated Methods section providing a more detailed account of the experimental procedures and of the different symbolic models used in the analyses.

To keep the response concise and focused, we address below the main comments raised by the reviewers. Minor remarks that led to straightforward corrections in the manuscript have been implemented directly and are not all listed individually.

Reviewer #1: In this manuscript, the authors investigate the alignment between human behavior and artificial neural networks in geometric shape perception across different model architectures and hyperparameter choices. Specifically, they assess the alignment between vision transformers (ViTs) and convolutional neural networks (CNNs), varying in number of parameters, training methods, and training set size, and human performance across three visual reasoning tasks of increasing complexity. Model performance is further compared with that of symbolic models used as baseline, which represent a low-dimensional set of rules capturing abstract relationships among geometric shapes. In Experiments 1 and 2, the authors find that artificial neural networks show greater correspondence with human behavior compared to the symbolic model, with the size of the pretraining dataset emerging as the primary factor driving this alignment. In Experiment 3, although artificial neural networks do not surpass the symbolic model, they nonetheless exhibit significant correspondence with human behavior, primarily influenced by training methodology and followed by training set size. Together, while acknowledging persistent gaps between artificial neural networks and human perception, the authors argue that these models—particularly when trained on large-scale datasets—offer a useful computational framework for probing the mechanisms underlying abstract visual reasoning.

---

1. Major comments:

(1.1) The Introduction suggests that the authors aim to compare the alignment of symbolic and deep learning–based AI models with human geometric shape perception. However, the manuscript does not provide a clear definition of what is meant by "symbolic," nor does it explain how symbolic models are conceptually related to the language-of-thought framework and how this differs from deep learning–based approaches.

Thank you for the remark. We added this definition in the introduction: "By symbolic, we refer to representational systems composed of discrete elements that can be composed into complex expressions according to a set of explicit rules, a grammar." (page 3 lines 27-29)

Convolutional neural networks (CNNs) and vision transformers (ViT) are known to learn high-level, human-relevant structure (e.g., concept detectors, objectness, or segmentation-like signals), raising the question of how such learned representations differ from what the authors term "symbolic." Indeed, one could argue that certain neural networks trained on large-scale datasets may develop proto-symbolic representations, which complicates a strict dichotomy between symbolic and neural models. For example, Thompson et al. (2024) showed that a relatively simple dual-stream recurrent neural network can encode the abstract relational structure between objects and generalize zero-shot to novel object types–both hallmarks of symbolic cognition.

If both approaches are capable of encoding high-level abstract information, the basis on which they

are being distinguished in the present study requires further clarification. If the primary distinction is simply that symbolic models rely on hand-coded representations whereas neural networks learn distributed representations through training, then the conceptual relevance of comparing symbolic and deep-learning models becomes less clear. Clarifying these distinctions and explicitly situating both modeling approaches within their respective theoretical frameworks would strengthen the conceptual grounding of the paper and help readers better understand the motivation for the comparisons made between ANNs and symbolic models.

Neural networks learn distributed representations; by design, they do not make use of explicit hand-coded symbols like the symbolic model. This, however, does not preclude the emergence of what the referee would call proto-symbolic representations. This is what we discuss p.21 lines 12-16: "Symbolic frameworks […] should not be understood as competing with neural implementations but as formal descriptions of the underlying computational logic. Neural networks may thus instantiate, in a distributed and implicit manner, the representational structures that symbolic theories make explicit, much like biological brains might do."

We believe that the result from Thompson et al. (2024), cited by the reviewer, reinforces our argument: a neural network can account for behaviors previously thought to require symbolic machinery — in their case, counting; in ours, geometry. The conceptual value of comparing both modeling approaches lies precisely in asking whether neural networks can capture the same abstract structure that symbolic models make explicit, and to what degree. We have expanded the introduction (p. 3, lines 38–42) to better situate this debate.

(1.2) It is also important to note that the symbolic models employed across the three experiments differ substantially in their assumptions and implementations. As a result, while it is informative to assess whether ANNs outperform a given symbolic model within a specific experiment, comparisons across experiments do not support general conclusions about whether symbolic models or ANNs better approximate human geometric shape judgments. In principle, a different or more nuanced instantiation of a symbolic model could capture human judgments equally well or even outperform ANNs. This raises questions about whether the specific symbolic models employed here provide a meaningful benchmark for assessing alignment with human geometric perception. Moreover, the manuscript offers limited discussion of the symbolic models' results and their comparison with ANNs. The authors may therefore wish to reconsider whether including symbolic models as a baseline is informative in this context, or alternatively, to more clearly articulate the intended role and interpretive value of symbolic models within the study as suggested in the previous comment.

We agree that the symbolic models vary across experiments and that comparisons across experiments do not support general claims about symbolic vs. neural approaches. We also agree that a more nuanced symbolic model could, in principle, perform better.

That said, the symbolic models used here do provide a meaningful baseline: they have been shown in prior work to capture human behavior well (Sablé-Meyer et al., 2021, 2022, 2025). We have added more information in the Methods section to explain better what are the symbolic models, and why they present a good, although imperfect, model of human behavior (p. 22, line 43 to p. 23 line 6, p. 23 line 49 to p. 24 line 16)

The key finding here is not that neural networks outperform all conceivable symbolic models, but that they outperform models which were previously considered good accounts of human behavior. This is informative precisely because it suggests that modern networks may help bridge the gap between connectionist and symbolic theories of geometric shape perception,

something older models did not achieve. We have added a paragraph in the introduction (p. 4, lines 18–24) to make this interpretive framing explicit.

(1.3) One of the main findings of the manuscript is that the size of the training dataset primarily predicts the alignment between human behavior and computer vision models. However, training set size is confounded with several other factors that could likely enhance the correspondence between humans and ANNs (see Supplementary Table 1). Larger datasets are typically associated with different training objectives (e.g., supervised ImageNet vs. contrastive CLIP or self-supervised DINO/DINOv3), substantial differences in dataset identity and visual richness (e.g., ImageNet-1k vs. ImageNet-22k or large-scale web/video corpora), increases in model capacity, and variations in pretraining pipelines, optimizers, and preprocessing. As a result, the apparent advantage of larger training sets may reflect a combination of these factors rather than dataset size per se. This confound represents a substantial limitation of the study and complicates the interpretation that training set size is the primary factor of human–model alignment in geometrical shape perception. Although this issue does not admit an easy solution given the available model zoo, it calls for a more cautious interpretation of the results, stronger methodological controls (see next comment), and a more explicit discussion of the relevant confounding factors.

It is true that there are confounds present between the various characteristics of the different neural networks, and, as the reviewer noted, there are some confounds that can hardly be controlled for. We have implemented the mixed effect model suggested in the next comment, and spent more time in the discussion to properly acknowledge, and when possible address, these limitations (p. 18, lines 31-38)

(1.4) Relatedly, it would be important to clarify whether the ridge regression analysis was cross-validated. If the regression was fit on the full dataset without cross-validation, the results are better interpreted as descriptive rather than predictive, and reporting standardized coefficients and uncertainty estimates may be more informative than permutation importance alone. However, I recommend using cross-validated feature-importance estimates (e.g., permutation importance on held-out folds) to reduce optimistic bias. Depending on how alignment is computed, this could be implemented via leave-one-participant-out cross-validation, or if alignment is based on an aggregate human RDM, using bootstrap/cross-validation over stimuli (e.g., split-half or k-fold over images/conditions to build human and model RDMs).

We thank the reviewer for this suggestion. For Experiments 1 and 2 we implemented cross-validation over participants as suggested; Experiment 3 has no participants over whom to fold. Cross-validation over stimuli was considered but not feasible: Experiment 1 has only 11 shapes, making held-out subsets too small for stable estimates, and in Experiment 3 the rank-based measure is computed relative to the full stimulus set and cannot be evaluated on a subset.

I suggest complementing the current ridge regression analysis with a hierarchical linear mixed-effects model in which the dependent variable is each model's human–model RDM alignment (e.g., the Spearman correlation between the upper triangles of the RDMs). While this approach will not eliminate collinearity among predictors, it provides a useful robustness check to assess whether training set size remains the dominant predictor within clusters of related models and training approaches, and it quantifies how much variance is captured by group-level effects versus dataset size. In particular, mixed-effects modeling can explicitly account for clustered dependence by including random intercepts for grouping variables such as training method and architecture family

(e.g., (1|training method), (1|architecture), or potentially (1 + log(training set size) | training method) and (1 + log(training set size) | architecture)). This reduces the risk that systematic differences shared by models trained under the same pipeline or stimulus set are inadvertently attributed to dataset size, as the model can allocate variance to the appropriate group-level terms rather than forcing it into the fixed effects.

Thank you for the recommendation. We added the mixed-model analysis (see Results, p. 7 line 43 to p.8 line 5, p.11 lines 31-37, p. 15 lines 16-25), and train set size was still a significant predictor of alignment controlling for these group level effects.

(1.5) Training set size is closely correlated with the richness and diversity of the visual diet, as larger datasets typically contain more varied and semantically rich stimuli. Importantly, Conwell et al. (2024) show through controlled analyses that it is this representational richness, rather than dataset size per se, that primarily drives increased alignment between human and model representations. This constitutes a confound that can hardly be resolved using statistical tricks in the current study, and it should therefore be explicitly acknowledged and discussed by the authors.

This is a good point: as things stand, we cannot control for the distribution of images in the training set. We thank the referee for the Conwell et al reference, which is very relevant; we added it to the discussion (p.18 lines 21-29).

(1.6) A particularly interesting finding of the study is that, in Experiment 3, the authors show that ANNs exhibit sensitivity to pairings between photographs and geometric drawings. However, this result is not entirely novel, and the manuscript would benefit from citing prior related work. For example, Singer et al. (2022) demonstrated that generalization to abstracted images, such as drawings, can emerge in CNNs trained on natural images.

Indeed, it is an important reference that we missed, thank you for pointing it out.

---

2. Minor points and clarity:

Comments (2.1) to (2.5), (2.8) and (2.9) are about methods, as well as clarity of the text and the figures. We have adressed these issues by adding a Methods section, and followed the reviewers recommendations on clarity in both text and figures.

(2.6) Page 8 - Paragraph 1: The authors introduce the cumulative distraction method as an alternative to Campbell's approach (2024), which was noted to be sensitive to outlier distractors. However, it is unclear how the cumulative distraction method addresses this limitation. In particular, extreme distractors—those with very large or very small distances—would still contribute equally when distances are averaged, potentially reintroducing the same sensitivity to outliers. Please clarify how this method mitigates the influence of outlier distractors, or otherwise justify its advantages over Campbell's original approach.

We now explain this more thoroughly in the methods section, page 24: "The centroid is strongly influenced by distractors that are far from the target: a single highly dissimilar distractor can shift the centroid away from the target, thereby increasing the target–centroid distance. Yet such a distractor should contribute very little to task difficulty. A highly dissimilar distractor can be rapidly rejected and effectively ignored during visual search, and therefore should have little impact on reaction time. In contrast, a distractor that is very close to the target should substantially increase difficulty and lead

to longer reaction times. Because the centroid aggregates all distractors symmetrically, it overweights distant distractors and underrepresents the impact of highly similar ones.”

(2.7) The authors note a potential confound between luminance and image complexity and attempt to address this by regressing out target luminance from choice time. However, another plausible confounding factor is spatial frequency: more complex images typically contain higher spatial frequency content. The authors may therefore also wish to consider regressing out spatial frequency–related measures, but results should not be affected as spatial frequency will likely be highly correlated with luminance.

This is true. We added an analysis (figure S4) controlling for more confounding factors, including spatial frequency. As the referee correctly predicted, conclusions were unchanged

Reviewer #2: This paper reports computational modelling results from three studies, in each of which representations within several deep neural networks (DNNs) are compared to previously-collected human data. The three studies involve different aspects of shape processing:
- Experiment 1: representational similarity in DNN layers is compared to human perceived similarity of simple quadrilaterals (inferred through speed and accuracy at detecting an outlying shape in a display)
- Experiment 2: representational distance in DNN layers is compared to human response time at matching a shape to that in a delayed display, using variously complex shapes
- Experiment 3: recognition (inferred through representational similarity) in DNN layers is evaluated for matching photos, line drawings, or geometric cartoons of objects, all of which can be perfectly recognised by human observers.

The results add to the literature on shape processing in DNNs, and the work is to be commended for the large number, diversity, and recency of DNNs evaluated.

MAJOR COMMENTS

The main issue with the current version of the paper is that it is missing a "Materials and Methods" section. Not enough detail to replicate the studies is provided in the main text, and the Supplementary Information contains only a few additional tables and figures. PLoS Comp Biol format generally involves a Methods section at the end of the manuscript. Such a section should be added, including the following:
- Explain software and hardware used in evaluating models and analysing data
- Explain stimuli, e.g. number of stimuli, how they were generated, resolution and colour of images, etc
- Explain model testing procedure, e.g. image preprocessing, number and method of image augmentation used to derive the "prototypical" representation for each image
- Explain all human experiments in brief - how were images presented, how long for, and how was the task phrased?
- Explain data analysis in more detail, i.e. each step involved in extracting and averaging model RDMs, each step involved in turning human response data into RDMs (e.g. in Expt 1, did behavioural RDMs somehow include both accuracy and response time data?), how model and human RDMs were compared (e.g. it seems that individual participant RDMs were averaged together, and the group-average RDM was used as the target with which to correlate each model RDM? But this is not

explained explicitly.), and, in more detail, how noise ceilings were calculated .

Thank you for this comment. Following the referee's recommendation, we have added a dedicated Methods section to the manuscript, which we hope addresses most of the referee's concerns. For the sake of brevity, we did not reproduce all details of the behavioral experiments, as they have been described more extensively in previous publications (Sablé-Meyer et al., 2021, 2022).

I will likely have more methodological comments once the methods are clearer. For now, I am wondering about the reason for the prototype procedure (averaging representation over multiple image instances) used in Experiment 1? This seems likely to tilt the results in the networks' favour, by artificially creating a "representation" which abstracts away from image specifics. An argument could perhaps be made for averaging over multiple translated snapshots (to simulate human eye movements during a trial) but here the averaging is done over scale and rotation, not translation. Is this analogous to human data in some way, e.g. is the human data also averaged over multiple trials? Although this doesn't undermine the results of Experiment 1, since the same procedure was applied to all models, it may inflate human correspondence relative to other studies which generally use only a single image to measure the representation of a stimulus. The decision should at least be explained and its implications discussed.

We thank the reviewer for raising this point. The prototype procedure was chosen specifically to match the structure of the human behavioral data: participants' dissimilarity were defined across trials presented at varying orientations and scales, so the human RDM already reflects a kind of implicit averaging over those transformations. Constructing neural prototypes by averaging over the same set of orientations and scales makes the neural representation commensurable with the human signal, rather than artificially inflating correspondence. We have clarified this rationale in the newly added Methods section (p. 23 lines 10-18).

MINOR COMMENTS

Comments referring to clarity of text or figures have been edited out, after resolving the issues following the reviewer's recommendations.

- p10: Euclidean/L2 distance is used to capture stimulus similarity in DNNs, whereas Manhattan/L1 distance is used in the symbolic model. Please explain why, and whether results differ if the same distance metric is used for both.

The use of Euclidean distance for the neural networks follows common practice in the literature when assessing similarity in DNN representations (Kornblith et al., 2019; Kriegeskorte et al., 2008). We also tested alternative similarity metrics commonly used in this context, including cosine distance and correlation, and found that they did not qualitatively alter the results (see Figure S1).

For the symbolic model, we use the L1 distance because it directly corresponds to the number of geometric features along which two shapes differ. We have clarified this point in the Methods section. Because the feature vectors are binary (containing only 0s and 1s), the L1 and L2 distances are in fact identical: for each feature, $|x_i - y_i| = (x_i - y_i)^2$. As a result, both metrics yield exactly the same pairwise distances.

- p10: Move paragraph containing "we systematically evaluated various vision models..." higher up in this Experiment 1 section, and explain more precisely which models were evaluated. E.g. state the number of models of each type (convolutional, ViT, supervised, unsupervised), what data they were trained on, and how they were accessed (e.g. weights of open-source pretrained networks were downloaded from original repositories).

Thank you for the suggestion. We believe the current ordering of the paragraphs in the Experiment 1 section preserves the intended flow of the description. Following this comment and other related remarks, we have added a Methods section that now provides detailed information about the neural networks that were evaluated, including their architectural families, training paradigms, training datasets, and how the pretrained weights were accessed.

- Figure 2D - it would be helpful to label a few of the model datapoints in this plot, e.g. the best and worst performing in each class, and/or those picked out for further examination in 2E

We attempted to add labels to a subset of datapoints, but model names are sufficiently long that even a few labels occupied a disproportionate amount of space and compromised readability. We therefore opted to keep the figure as is.

- p14: "sum of the inverse distances" - I am not familiar with the term "inverse distance"; please explain how this differs mathematically from the sum of the distances.

The inverse distance is defined as 1 divided by the distance. This is now explained in more details in the Methods section.

- Experiment 3 should be discussed in the context of other papers that have examined representation and recognition of line drawings in DNNs, e.g. Kubilius et al. (2016), Fan et al (2018), Singer et al (2022)

Thank you for the comment, these are useful references that we now discuss (p. 17 lines 25-29).

Reviewer #3: This is an interesting and potentially valuable paper that compares how humans and DNNs represent abstract geometric shapes, extending past work on this topic. In particular, they take data from three human behavioral experiments, devise “DNN analogues” of these tasks, and compare the human data to many different DNNs, varying the architecture and training data. They also compare performance to a symbolic network that was successful in a previous study.

The manuscript nicely justifies the importance of the research question and it would indeed be an interesting finding if the newer wave of DNNs can better capture the abstract visual processing exhibited by humans than a symbolic model. However, in general I find that a bit more work is needed to convincingly demonstrate this.

SPECIFIC COMMENTS:

Comments 4, 6, 8 and 10 are about clarity of the text and figures, and the issues have been adressed following the reviewers recommendations.

1. It is currently impossible for the reader to understand the comparisons to the symbolic model without explaining it in more detail. In Experiment 1 it is a vector of quadrilateral properties. It isn't clear what the "symbolic model" corresponds to in Experiment 2. This raises a number of issues both at the level of prose clarity and the analyses that were performed:
a. A more specific label than "the symbolic model" might be nice just to establish that it is in fact the same model throughout the paper; it is confusing that it is introduced in the context of quadrilaterals in Experiment 1, but is clearly more broad in Experiment 2, and coexists alongside the MDL, which is also related to a symbolic model of the shapes.

Thank you for the comment. We added a Methods section, in which there is more information on the symbolic models. The models in Experiment 1 and 2 differ in nature: as the referee noted, it consists of a vector of properties in Experiment 1. In Experiment 2, however, the symbolic model that we use is the Minimum Description Length of the target shape, as defined according to the Language of Thought for geometric shapes in (Sablé-Meyer et al., 2022).

b. In computing the representational similarity of the shapes based on the symbolic model, it seems like all of the properties are weighted equally, which arguably loads the dice against the symbolic model. What if only a subset of the properties are used by humans in the task (and are sufficient to fully predict performance), with the rest only contributing noise? Especially given that these properties seem to be of different kinds ("symmetry", "right angles", "parallel lines"), it isn't clear that an unweighted L1 norm makes a lot of sense. Along the same lines, I take it Pearson correlation was used to compare RDMs—this at the very least requires justification (why not use Spearman?), since human reaction times and DNN activation distances are rather different quantities.

The equal weighting of properties follows the original formulation of the model (Sablé-Meyer et al., 2021, 2025), where it provided a strong account of human behavior. We have clarified and justified this choice in the Methods section (p. 22 line 43 to p. 23 line 6).

Regarding the use of Pearson correlation: this follows standard RSA practice, in which Pearson correlation is commonly used to compare RDMs across representational spaces of different natures. The purpose of RSA is precisely to enable such comparisons via the structure of pairwise dissimilarities, abstracting away from the specific scale or type of the original measurements. That said, we have verified that Pearson and Spearman are highly consistent across models (Supplementary figure S3), and mention it in the text (p. 7 lines 1-2).

c and d. To derive the "prototype patterns" for each quadrilateral, they took the average embedding across different scales and orientations. I assume this was not also done for the symbolic model because the properties in the symbolic model are already scale/rotation invariant (something else that would be nice to know). I additionally assume that this is meant to reflect the actual outlier detection task, in which the distractor shapes also varied based on their scale and orientation. While this seems fair from one perspective, one might also argue that it's loading the dice in favor of DNNs: averaging out all the scale- and orientation-specific features will obviously increase the prominence of the abstract, invariant features in the prototype embedding, further complicating the comparison between the DNNs, the symbolic models, and the human data (and after all, this "prototype" is a statistical abstraction that doesn't arise from any real single input to the DNN). Further justification

for these analysis steps is needed. This prototype-averaging procedure also complicates the later analysis where they look at how low-level visual features contribute to the results—doesn't averaging across scales and orientations throw out many of these low-level features? Again, more justification would be nice.

The reviewer is correct on both counts. The symbolic model does not require this averaging step because its properties are defined analytically and are therefore invariant to scale and rotation by construction — we have clarified this in the text (p. 23 lines 16-18). The averaging for DNNs is not intended to favor any particular model class, but, as the reviewer noted, to match the structure of the human data: participants judged shapes across trials that varied in orientation and scale, so the prototype reflects the same kind of summary that underlies human behavioral responses.

We acknowledge the reviewer's concern — that averaging could be seen as artificially amplifying invariant features in DNN representations — but argue that the alternative (using a single arbitrary orientation and scale) would introduce an arbitrary and potentially more consequential bias. The procedure was applied symmetrically across all DNN models, so relative comparisons between networks are unaffected.

Regarding the concern about low-level visual features, averaging does not eliminate them altogether, but only reduces variation tied to specific orientations or scales. Features that are stable across these transformations remain present in the representations and can still contribute to the analysis.

2 and 9. It was not clear why these three tasks were chosen. I assume these are not the only behavioral tasks measuring the similarity of abstract shapes; if there's particular reason for using data from these experiments over others it'd be nice for it to be spelled out. The logical relationship of Experiment 3 to the other two experiments is a bit unclear—we have lost the symbolic model that seemed key to the other two experiments and the stimuli are quite different. Again, some rationale for why these three experiments were chosen could be helpful.

We added this paragraph to the introduction (p.5 lines 12-16): "Together, these three tasks probe the ability of neural networks to capture human geometric cognition across increasing levels of complexity. Experiment 1 examines shape representations in a narrowly controlled setting, the perception of quadrilaterals; Experiment 2 extends this to a broader repertoire of non-figurative geometric shapes. Experiment 3 turns to the perception of abstract figurative drawings, another hallmark of human shape cognition."

3. A bit more clarity in the introduction would be nice, as the words "abstract" and "geometric" can be interpreted different ways. It's eventually clear enough what the authors mean, but a few examples (akin to the intro of Experiment 3) could help the reader zero in more quickly.

Thank you for the comment. We added this definition in the introduction (p.3 lines 5-7): "Here, we use this term "geometric shape" to refer both to non-figurative shapes that consist of simple lines and curves organized by spatial regularities, and to schematic figurative drawings depicting objects or scenes using such geometric elements"

5. In Experiment 1 it looks like you are using a Pearson correlation coefficient as the DV in multiple regression. Strictly speaking I believe this isn't kosher (since Pearson correlation is bounded between

-1 and +1) and violates the assumptions of linear regression. I think it's quite plausible this won't matter, but this needs to be addressed in some way.

This is a good catch. We now apply the Fisher z-transform to the correlation values before entering them as the dependent variable in the ridge regression, in both Experiments 1 and 2. This ensures the dependent variable is unbounded and variance-stabilized, satisfying the assumptions of linear regression. Results were unchanged.

7. In Experiment 2, you nicely regress out mean luminance as a potential confound with the complexity of the shape. But aren't there other such confounds too (area, number of edges, etc.)? I would need more convincing that luminance is the only confound of this kind.

There are indeed more possible confounds that are correlated with the Minimum Description Length. We added an analysis with all the identified confounds in (Sablé-Meyer et al., 2022) and results were unchanged (p. 11 lines 9-11, Supplementary figure S4).

11. The discussion is theoretically rich, but would benefit from a more thorough comparison between the symbolic model and the DNNs, as noted earlier, and the claims about a "Language of Thought" seem like a bit of a stretch from the actual findings of the paper.

We appreciate the reviewer's engagement with the theoretical discussion. Regarding the symbolic model comparison, following this remark and others by the reviewers we have expanded the introduction to include additional discussion of the symbolic model, which we believe addresses this concern without overburdening the discussion.

On the Language of Thought, we stand by our interpretation. We are careful to present the LoT framing as one of two contrasting interpretations, explicitly acknowledging the alternative view that the success of large-scale neural networks renders symbolic theories unnecessary or of limited utility. The interpretation we favor, that symbolic frameworks serve as high-level descriptions of computations implemented by neural systems, is positioned as a theoretical perspective rather than a direct empirical claim, and we have ensured the relevant passages make this distance clear.
