# Supplementary material for: Can neural networks model the human perception of geometric shapes?
Source: PLoS Comput Biol. 2026 Jul 27;22(7):e1014554. doi: 10.1371/journal.pcbi.1014554 (PMC13440860; doi:10.1371/journal.pcbi.1014554)
Supplement: Supplementary file 4 [file pcbi.1014554.s010.docx]

Reviewer #1:

(1.1) The authors now provide a precise definition of a symbolic model in the Introduction.

While technically accurate, the definition remains quite abstract and may be difficult or a broader readership to grasp. Since the manuscript draws comparisons with connectionist approaches, it would be helpful to include a parallel definition of a connectionist model that mirrors the structure of the symbolic model definition.

For example, a connectionist model could be described as a system that learns distributed representations by optimizing continuous connection weights, allowing complex patterns to emerge without explicitly specified rules, beyond the implicit constraints imposed by the architecture and training setup. Presenting both definitions in a comparable format would help clarify the key distinctions between symbolic and connectionist models and would also provide an implicit contrast (i.e., a definition by negation) of symbolic models.

Thank you for the recommendation. Following the reviewer’s suggestion, we added these sentences (p. 4 lines 2-5):

“In contrast to symbolic systems, a connectionist model processes information through distributed patterns of activation across a network of simple units. Rather than relying on discrete representations and explicit rules, it learns continuous-value connection weights to capture complex patterns, relying only on the constraints of its architecture and training.”

(1.2) I appreciate the authors’ clarification and the expanded discussion. However, I remain unconvinced by the claim that the present findings “bridge the gap” between connectionist and symbolic accounts, particularly given the limited understanding of the mechanisms underlying human geometric shape perception. Demonstrating that modern ANNs better approximate human behavior than prior symbolic models does not necessarily imply that these ANNs implement more “symbolic-like” representations or that both approaches converge onto similar representational structures, as mentioned in the Discussion.

We thank the reviewer for highlighting this point, which has helped us identify where our framing could be made clearer. We want to clarify that we do not claim modern ANNs bridge connectionist and symbolic accounts, nor that they implement symbolic-like representations. While this 'bridging' language was used for conceptual context in our previous response letter, it is not a claim made in the manuscript itself. In the paper, we simply raise the possibility that ANNs might learn representations with certain symbolic-like properties as one of several interpretations, flagged explicitly as a direction for future work. To ensure there is no ambiguity for the reader, we propose adding a clarifying statement to the Introduction section to explicitly bound the scope of these interpretations and rule out any unintended claims of a structural ‘bridge’ (p.4 lines 17-19):

”We do not try to adjudicate between symbolic and connectionist accounts of geometric cognition, and pursue a more tractable goal: characterizing the conditions under which neural networks representations align with humans on geometric tasks”

Relatedly, the connection between the symbolic models used in this study and the language-of-thought framework remains unclear. While this link is suggested in the Introduction, it is not explicitly specified. It would be helpful for the authors to clarify whether these symbolic models are theoretically grounded in the language-of-thought hypothesis or instead constitute ad hoc, rule-based formulations. If the latter, their contribution requires further clarification. In particular, since the current symbolic models were human-designed, it is perhaps unsurprising that they align with human behavior better than old CNN models.

We have now made the link to the language-of-thought framework explicit in the Introduction (p. 3, lines 29–34), describing the models of Sablé-Meyer et al. (2021, 2022) as formal instantiations of LoT applied to geometric perception: compositional programs built from a small set of primitives under an explicit grammar. They are not ad hoc rule systems.

While the manuscript convincingly shows that modern ANNs provide increasingly human-like representations, the added value of including symbolic models in the current manuscript to advance our understanding of human shape perception or ANN representations is currently unclear.

The role of the symbolic models in this study is to provide a theoretically motivated baseline, grounded in an independent framework (LoT). We believe this baseline is informative for two reasons. First, symbolic models had outperformed earlier neural networks, and assessing whether modern architectures have closed this gap is, in itself, a useful empirical update. Second, the comparison helps characterize what modern ANNs do well and where they still fall short. We explain this in the introduction (p. 5, lines 12-14):

“As a baseline, we compared these neural networks to the symbolic models introduced by Sablé-Meyer et al. (2021, 2022). In our previous work, these models captured human behavior better than earlier neural networks, making them a meaningful benchmark for assessing whether modern neural networks now match or exceed symbolic accounts of geometric cognition.”

(1.7) I note that the noise ceiling estimates in Experiment 1 are very high (r = 0.99).

The noise ceiling was computed by pooling behavioral measurements across participants within each split iteratively. This means that noise was effectively cancelled across 165 participants within each split. If we assume that each participant can be treated as a distinct model, this procedure effectively removes inter-model (i.e., between-subjects) noise. For ANNs, however, measurements were averaged within each model across different presentations of the same shape, which primarily removes intra-model noise (i.e., within-subject). Thus, although averaging was applied in both cases, the type of noise being cancelled differs between humans and models. This asymmetry may result in an artificially high noise ceiling, effectively setting an overly optimistic benchmark for ANN performance.

This is a critical point that may also affect Experiment 2 and should be addressed more explicitly. One possible approach would be to estimate the noise ceiling using a leave-one-subject-out procedure. On each iteration, a single participant is held out, and an RDM is computed from the remaining (n–1) participants. A second RDM is computed for the held-out participant, and the noise ceiling is estimated as the average correlation between each participant and the corresponding group-level RDM. This would provide a more comparable estimate to the correlations obtained between ANN models and the group-level human RDM.

Thank you for this remark. The high noise ceilings in Experiments 1 and 2 (r = 0.99 and r = 0.90, respectively) reflect a deliberate methodological choice rather than an overestimation. Split-half reliability quantifies how reproducible the group-level RDM is across two halves of the data, and as such provides a meaningful upper bound on what any model could possibly explain when compared against that group average.

The reviewer's concern rests on the assumption that individual participants can be treated as distinct models. We would argue this is not the case in the case of our short on-line experiments, where we privileged brevity and collected relatively few data per participants, in order to obtain a large number of volunteers. As a result, single-participant RDMs were highly noisy, and human similarity judgments became much more stable and interpretable once averaged across many trials and many participants. The split-half procedure respects this by operating on aggregated data, which is precisely the quantity our ANN models are being compared against.

To validate this, we also computed new noise ceilings using the suggested leave-one-subject-out approach, correlating each held-out participant's RDM with the group RDM formed from the remaining participants. This yields noise ceilings of 0.77 and 0.41 in Experiments 1 and 2 respectively, values that fall well below the performance of our best-performing models. Rather than providing a more conservative and appropriate benchmark, this procedure produces a noise ceiling that underestimates the true reliability of the data, because it conflates measurement noise at the individual level with the reliability of the group representation we actually seek to model.

We added a note on noise ceiling estimation in the Supplementary materials for the interested reader, and a sentence in the Methods section to refer to it (p. 22 line 28)

(1.8) While the authors’ efforts to improve the Introduction and Discussion have strengthened the manuscript, the overall presentation still lacks clarity. In particular, the Introduction remains somewhat difficult to follow and, at times, potentially misleading, primarily due to the absence of a clear and coherent narrative guiding the reader through the paper’s objectives.

The manuscript begins by introducing geometric shape perception and then motivates the idea that human shape perception may be symbolic, drawing on the language-of-thought framework and situating the work within the renewed debate between connectionist and symbolic models. At this stage, the reader may reasonably infer that the primary goal of the manuscript is to arbitrate between these two accounts. However, the subsequent paragraph shifts focus to identifying which ANN hyperparameters yield greater human alignment at the behavioral level. The manuscript then returns to symbolic models as a baseline, framing the goal as evaluating whether contemporary neural networks can match or exceed symbolic models previously shown to capture human behavior. This is followed by the introduction of CNNs and ViTs, further broadening the scope.

Overall, the progression of ideas lacks a clear through-line, and the specific research question and contribution of the manuscript remain somewhat ambiguous. I believe the manuscript would benefit from substantial revision to more clearly define its goals, better situate the theoretical context, and improve the organization of both the Introduction and Discussion. Strengthening the narrative structure would make the paper significantly easier to follow and interpret.

We thank the reviewer for this remark. We have rewritten the Introduction to make the through-line clearer. The revised Introduction states our two contributions explicitly in a dedicated paragraph: first, testing whether modern neural networks align with human behavior on geometric shape tasks, and how they compare to symbolic models; second, identifying which of their properties drive that alignment.

We first introduce the context of the study as before, presenting geometric shape cognition and symbolic and connectionist models. We then explicitly state our contributions in the dedicated paragraph mentioned above. We end the Introduction with a brief overview of the experiments, the networks we tested, and the main results.

We have also shortened the description of the CNNs and ViTs to keep the Introduction focused on the study's framing and contributions.

(1.9) I believe the Discussion would benefit from considering the possibility that the alignment between modern ANNs and humans is driven by high-level visual features. This interpretation is consistent with prior work suggesting that shape perception may rely on abstract structural properties of objects (e.g., shape skeletons; Chaz Firestone & Brian Scholl, 2014; Valeria Ayzenberg & Stella

Lourenco, 2022).

This is an interesting suggestion, we added the following paragraph to the discussion to address it (p. 19, lines 25-30):

“One possibility is that this shared representation relies on high-level structural features of shape, such as shape skeletons, which have been proposed to underlie human shape perception (Ayzenberg & Lourenco, 2019; Firestone & Scholl, 2014). However, this account seems unlikely to fully explain our findings: Sablé-Meyer et al., (2026) reported that skeletal descriptions provided a poor fit to human behavior on quadrilateral perception, the very task in which we observe the strongest alignment between networks and humans.”

(1.10) I encourage the authors to include a dedicated Data Availability section within the Methods. This section should explicitly state whether the data are openly available and, if so, provide hyperlinks and clear information on how they can be accessed. Doing so would align the manuscript with the open science principles of PLOS Computational Biology and enhance transparency and reproducibility.

We agree that open data and code is an essential part of research, to which we are committed. PLOS Computational Biology has a dedicated “Data and Code availability” segment, outside of the Methods section, in which the availability of both data and code is stated as such:

“All data and code are available at the following GitHub repository: https://github.com/MaxencePajot/geometry_in_neural_networks”

(2.10) Page 3, line 38: The statement that recent advances in AI have rekindled thedebate between classical and connectionist theories would benefit from the inclusionof recent references explicitly engaging with this debate. This would help situate the current work within the contemporary literature.

We completed the sentence as such in the introduction (p. 3 line 42 to p. 4 line 2)

“Recent advancements in artificial intelligence have rekindled the debate between classicists and connectionist theories of cognition (Fodor & Pylyshyn, 1988; Smolensky, 1988), with some arguing that the success of large neural networks challenges the usefulness of symbolic theories (Griffiths et al., 2025; Piantadosi, 2023), while others maintain that symbolic descriptions remain indispensable (Dehaene, 2018; Dehaene et al., 2022; Katzir, 2023)

(2.11) Figure 1B: The graphical representation of the symbolic model is somewhat confusing. While it appears intended to depict a syntactic tree structure, representing it as a set of interconnected nodes resembles a connectionist architecture and may blur the intended distinction. Consider revising the figure.

Thank you for this point, we see how the symbolic model’s representation might be confusing. We replaced it with a figure with clearer emphasis on the hierarchical tree structure to alleviate the similarity to a connectionist model.

(2.14) Page 7: The claim that dataset size is the dominant predictor of behavioral alignment is not supported with sufficient statistical detail. Please report relevant statistics (e.g., effect sizes, confidence intervals) to convey the magnitude of the effects. Additionally, clarify whether the permuted feature importance distributions were directly compared across variables.

We added the average drop in performance across permutations for the relevant variables (p. 7 lines 41-45, p. 11 lines 27-28, p. 15 lines 5-6). The distributions are directly compared in the revised text: dataset size (ΔR² = 0.84 ± 0.18) does not overlap with any other predictor (next largest: architecture, ΔR² = 0.23 ± 0.08), indicating the difference is well outside the range of permutation noise.

(2.16) Page 12, line 15: The specific distance metric should be explicitly stated in the Methods section. If Euclidean distance was used as mentioned in the Results section, it would be helpful to note that it can be influenced by the dimensionality of the feature space, with higher dimensionality typically leading to larger distances. While this may not critically affect the construction of RDMs, acknowledging this point—and ideally confirming that similar results are obtained with alternative distance measures less sensitive to dimensionality (i.e., cosine distance)—would strengthen the analysis.

We added these sentences to the Methods section (p. 26 line 44 to p.27 line 2):

“We used Euclidean distance to measure distances between embeddings. Equivalent results were obtained with cosine distance and correlation (Supplementary figure S1), which are less sensitive to dimensionality.”

Additionally, please specify how RDMs were compared (e.g., Spearman correlation) to improve clarity and reproducibility.

This sentence was added to the methods section (p. 23 lines 21-22):

“Alignment between model RDMs and behavioral RDMs was quantified using Pearson correlation.”

Reviewer #2: The authors have done an impressive amount of work in this revision. They have added an extensive and informative Materials and Methods section in response to my request for one (and several other methods clarification questions from other reviewers). And they have engaged thoughtfully with the various critiques and suggestions raised in review. Methodological decisions that I found puzzling in the first submission (e.g. averaging DNN activations over multiple presentations of a stimulus) are well justified now that further detail has been provided about the human experimental design. I find the revised paper interesting and well presented and have no further comments.

Thank you!

Reviewer #3: I thank the authors for their thoughtful changes to the manuscript in response to reviewer comments. The added methods section and details about the symbolic models have greatly improved clarity, and the supplementary statistical analyses (e.g., using spearman instead of Pearson; applying Fisher Z-transform to the correlation values prior to the regression analysis; controlling for low-level factors in addition to luminance) have done much to shore up the sturdiness of these findings. Additionally, the prose-level changes have nicely improved the clarity of the manuscript. That said, there remains one major issue I would like to see addressed. Both I and another reviewer raised the point that averaging the DNN embeddings across variation in scale and angle in Experiment 1 might artificially tilt the scales towards finding evidence of “symbolic” representations in DNNs. The authors responded that the human data is also aggregated across variation in scale and angle, so this averaging step remains valid.

However, this response elides a subtle but crucial distinction. In the human case, shapes with a particular size/orientation are presented, and the time/accuracy for finding the “odd one out” is measured. These behavioral measures—which constitute the measured dissimilarity for the purposes of RSA—are collected in an experimental setting that includes size/orientation variation, and are only subsequently averaged.

By contrast, for the DNNs the embeddings for different sizes/orientations are averaged first, and the dissimilarity is computed afterward. These forms of averaging are not the same: for humans dissimilarity is measured between specific exemplars of each shape and averaged afterwards (i.e., average distance between specific exemplars), and for DNNs dissimilarity is computed between constructed statistical “prototypes” that don’t correspond directly to any actual input seen by the DNN (i.e., distance between average of exemplars). The truly analogous procedure would be: for shapes A and B, feed all size/orientation variants of A and B into the DNN, compute the distance between each variant of A and each variant of B (taking all combinations), and only then take the average distance. It is easy to see that these procedures will yield different measured dissimilarities: given two multivariate Gaussians, the distance between random draws of those Gaussians will in general be bigger than the distance between their centroids. Furthermore, this distinction is of theoretical interest: it could be the case that DNNs contain size/orientation invariant visual information (as they almost surely do), but that this information plays a smaller role in the representation than the “nuisance” variables that are discarded in the averaging procedure.

The authors are correct that they apply the same procedure to all DNNs, so this issue doesn’t apply to the between-DNN comparisons, but since the comparison between DNNs and symbolic models is foundational to the manuscript, this point simply must be addressed to warrant the authors’ comparative claims.

Thank you for this recommendation. We agree that this is a more rigorous way to assess the representations of quadrilaterals in DNNs, and have now implemented it in place of our prototyping method in experiment 1. The results change slightly, but the main conclusions remain. While we observe an overall drop in performance across the board, especially for CNNs, the best networks still perform substantially better than the symbolic model (~0.9 correlation with behavior), and the main factor driving alignment is the size of the training dataset.
