# Supplementary material for: Can neural networks model the human perception of geometric shapes?
Source: PLoS Comput Biol. 2026 Jul 27;22(7):e1014554. doi: 10.1371/journal.pcbi.1014554 (PMC13440860; doi:10.1371/journal.pcbi.1014554)
Supplement: Supplementary file 5 [file pcbi.1014554.s011.docx]

1. Introduction:
-Page 1, second paragraph: The authors begin with the phrase “In line with the Language of Thought Hypothesis”, but the hypothesis has not yet been introduced. It would be preferable to first define the Language of Thought Hypothesis and then refer back to it.

We modified this part of the introduction to better link symbolic systems and the language of thought hypothesis :

“By symbolic, we refer to the representational format posited by the language of thought hypothesis: systems composed of discrete elements that can be composed into complex expressions according to a set of explicit rules, a grammar”

-Page 1, third paragraph: The manuscript refers to “classicist theories of cognition”, but this term is not clearly defined. I would personally avoid using the term “classicist”, because given the long history of connectionist models, some readers may also view them as “classical” theories in a broader sense.

We replaced “classicist” with “symbolic”.

-Page 2: While I understand the authors’ intention, the following sentence reads somewhat awkwardly: “We do not try to adjudicate between symbolic and connectionist accounts of geometric cognition, and pursue a more tractable goal.” Because the second clause contrasts with the first rather than simply adding information, a construction using a contrastive expression (e.g., “instead” or “rather than”) may better convey the intended meaning than the copulative conjunction “and”.

We changed the sentence as follows:

“We do not try to adjudicate between symbolic and connectionist accounts of geometric cognition, but rather pursue a more modest and tractable goal:”

-The Introduction states twice that the symbolic models of Sablé-Meyer et al. (2021, 2022) are used as baselines. This point only needs to be made once and could be streamlined.

We deleted the first instance of this point.

2. Figure 1: The authors indicate that the image representing the symbolic model has been modified; however, the figure appears unchanged from the previous version. In addition, Figure 1 currently lacks a descriptive caption header.

This was a mistake on my part, and I apologize for the confusion. The issue has been fixed.

This header was added for Figure 1: “Figure 1: Geometric stimuli and modeling framework to evaluate geometric shape perception in neural networks.”

3. Noise ceilings: I understand the authors’ rationale for their approach to estimating noise ceilings, although it may be considered somewhat unorthodox. I do not intend to revisit this issue in a third round of review. Nevertheless, given the central importance of noise ceilings in a correlational study of this kind, I believe the authors should explicitly acknowledge the relatively low between-subject reliability of the behavioral data as a limitation of the current study in the Discussion.

We added this paragraph to the Discussion (p. 19. Lines 32-38):

“Even though the networks accurately modelled human behavior in the first task, they left some variance unexplained in the next two tasks. Specifically, the aggregated group-level pattern of results used as our modeling target was highly reliable, as judged from its split-half noise ceiling, and yet the models did not achieve to fully capture all of this explainable variance. (We note in passing, as a limitation, that we focused solely on group-average results; indeed, the reliability of individual judgments was relatively low, mostly due to a small number of within-subject trials, and prevented us from studying inter-individual variability; see Supplementary materials for discussion).“

4. Abstract: The authors refer to a “language of geometry” in the Abstract, but this term does not appear elsewhere in the manuscript. To avoid potential confusion, I would suggest either removing the term from the Abstract or introducing and defining it in the Introduction. If retained, it may fit naturally alongside the introduction of the Language of Thought hypothesis (see comment above).

The language of geometry was presented in the introduction, yet without explicitly using this term. We changed it as follows (p.3 line 33):

“The mental representation of a shape would be encoded as a “mental program” in this internal language of geometry: a compositional expression that draws the shape by combining a small set of primitives according to the grammar’s rules.”
