# Supplementary material for: Can neural networks model the human perception of geometric shapes?
Source: PLoS Comput Biol. 2026 Jul 27;22(7):e1014554. doi: 10.1371/journal.pcbi.1014554 (PMC13440860; doi:10.1371/journal.pcbi.1014554)
Supplement: Peer review history

# Peer review: Decision Letter 0

**DOI:** 10.1371/journal.pcbi.1014554.r001

**Authors:** Lyle Graham, Alejandro Tabas

26 Jan 2026

PCOMPBIOL-D-25-02201

Can Neural Networks model the human perception of geometric shape

PLOS Computational Biology

Dear Dr. Pajot,

Thank you for submitting your manuscript to PLOS Computational Biology. After careful consideration, we feel that it has merit but does not fully meet PLOS Computational Biology's publication criteria as it currently stands. Therefore, we invite you to submit a revised version of the manuscript that addresses the points raised during the review process.

Please submit your revised manuscript by Mar 28 2026 11:59PM. If you will need more time than this to complete your revisions, please reply to this message or contact the journal office at ploscompbiol@plos.org. When you're ready to submit your revision, log on to https://www.editorialmanager.com/pcompbiol/ and select the 'Submissions Needing Revision' folder to locate your manuscript file.

Please include the following items when submitting your revised manuscript:

* A letter that responds to each point raised by the editor and reviewer(s). You should upload this letter as a separate file labeled 'Response to Reviewers'. This file does not need to include responses to formatting updates and technical items listed in the 'Journal Requirements' section below.

* A marked-up copy of your manuscript that highlights changes made to the original version. You should upload this as a separate file labeled 'Revised Manuscript with Track Changes'.

* An unmarked version of your revised paper without tracked changes. You should upload this as a separate file labeled 'Manuscript'.

If you would like to make changes to your financial disclosure, competing interests statement, or data availability statement, please make these updates within the submission form at the time of resubmission. Guidelines for resubmitting your figure files are available below the reviewer comments at the end of this letter

We look forward to receiving your revised manuscript.

Kind regards,

Alejandro Tabas, Ph.D.

Academic Editor

PLOS Computational Biology

Lyle Graham

Section Editor

PLOS Computational Biology

**Additional Editor Comments:**

Many thanks for you submission and your work. Two of the reviewers warned us that they may recommend rejection after the first round of reviews. If both of them do, we will probably have to reject the paper at that stage. I wanted to let you know about this possibility before you invest time in the revision, although we would of course be very happy to receive a revised version of the MS.

**Journal Requirements:**

If the reviewer comments include a recommendation to cite specific previously published works, please review and evaluate these publications to determine whether they are relevant and should be cited. There is no requirement to cite these works unless the editor has indicated otherwise.

1) Your manuscript is missing the following sections: Results, and Methods.  Please ensure all required sections are present and in the correct order. Make sure section heading levels are clearly indicated in the manuscript text, and limit sub-sections to 3 heading levels. An outline of the required sections can be consulted in our submission guidelines here:

https://journals.plos.org/ploscompbiol/s/submission-guidelines#loc-parts-of-a-submission

2) Please upload all main figures as separate Figure files in .tif or .eps format. For more information about how to convert and format your figure files please see our guidelines:

https://journals.plos.org/ploscompbiol/s/figures

3) We have noticed that you have uploaded Supporting Information files, but you have not included a list of legends. Please add a full list of legends for your Supporting Information files after the references list.

4) 4A includes an image of an identifiable person. Please provide written confirmation or release forms, signed by the subject(s) (or their guardian), giving permission to be photographed and to have their images published under a Creative Commons license. You may upload permission forms to your submission file inventory as item type 'Other'. Otherwise, we kindly request that you remove the photograph.

5) Some material included in your submission may be copyrighted. According to PLOSu2019s copyright policy, authors who use figures or other material (e.g., graphics, clipart, maps) from another author or copyright holder must demonstrate or obtain permission to publish this material under the Creative Commons Attribution 4.0 International (CC BY 4.0) License used by PLOS journals. Please closely review the details of PLOSu2019s copyright requirements here: PLOS Licenses and Copyright. If you need to request permissions from a copyright holder, you may use PLOS's Copyright Content Permission form.

Please respond directly to this email and provide any known details concerning your material's license terms and permissions required for reuse, even if you have not yet obtained copyright permissions or are unsure of your material's copyright compatibility. Once you have responded and addressed all other outstanding technical requirements, you may resubmit your manuscript within Editorial Manager.

Potential Copyright Issues:

i) Please confirm (a) that you are the photographer of 4A, or (b) provide written permission from the photographer to publish the photo(s) under our CC BY 4.0 license.

ii) Figures 1, 3A, and 4A. Please confirm whether you drew the images / clip-art within the figure panels by hand. If you did not draw the images, please provide (a) a link to the source of the images or icons and their license / terms of use; or (b) written permission from the copyright holder to publish the images or icons under our CC BY 4.0 license. Alternatively, you may replace the images with open source alternatives. See these open source resources you may use to replace images / clip-art:

- https://commons.wikimedia.org

- https://openclipart.org/.

6) Please amend your detailed Financial Disclosure statement. This is published with the article. It must therefore be completed in full sentences and contain the exact wording you wish to be published.

1) State what role the funders took in the study. If the funders had no role in your study, please state: "The funders had no role in study design, data collection and analysis, decision to publish, or preparation of the manuscript."

2) If any authors received a salary from any of your funders, please state which authors and which funders..

If you did not receive any funding for this study, please simply state: u201cThe authors received no specific funding for this work.u201d

**Reviewers' comments:**

Reviewer's Responses to Questions

**Comments to the Authors:**

**Please note here if the review is uploaded as an attachment.**

Reviewer #1: In this manuscript, the authors investigate the alignment between human behavior and artificial neural networks in geometric shape perception across different model architectures and hyperparameter choices. Specifically, they assess the alignment between vision transformers (ViTs) and convolutional neural networks (CNNs), varying in number of parameters, training methods, and training set size, and human performance across three visual reasoning tasks of increasing complexity. Model performance is further compared with that of symbolic models used as baseline, which represent a low-dimensional set of rules capturing abstract relationships among geometric shapes. In Experiments 1 and 2, the authors find that artificial neural networks show greater correspondence with human behavior compared to the symbolic model, with the size of the pretraining dataset emerging as the primary factor driving this alignment. In Experiment 3, although artificial neural networks do not surpass the symbolic model, they nonetheless exhibit significant correspondence with human behavior, primarily influenced by training methodology and followed by training set size. Together, while acknowledging persistent gaps between artificial neural networks and human perception, the authors argue that these models—particularly when trained on large-scale datasets—offer a useful computational framework for probing the mechanisms underlying abstract visual reasoning.

----------------------------------------------------------------------------------------------------------------------------

1. Major comments:

(1.1) The Introduction suggests that the authors aim to compare the alignment of symbolic and deep learning–based AI models with human geometric shape perception. However, the manuscript does not provide a clear definition of what is meant by “symbolic,” nor does it explain how symbolic models are conceptually related to the language-of-thought framework and how this differs from deep learning–based approaches.

Convolutional neural networks (CNNs) and vision transformers (ViT) are known to learn high-level, human-relevant structure (e.g., concept detectors, objectness, or segmentation-like signals), raising the question of how such learned representations differ from what the authors term “symbolic.” Indeed, one could argue that certain neural networks trained on large-scale datasets may develop proto-symbolic representations, which complicates a strict dichotomy between symbolic and neural models. For example, Thompson et al. (2024) showed that a relatively simple dual-stream recurrent neural network can encode the abstract relational structure between objects and generalize zero-shot to novel object types–both hallmarks of symbolic cognition.

If both approaches are capable of encoding high-level abstract information, the basis on which they are being distinguished in the present study requires further clarification. If the primary distinction is simply that symbolic models rely on hand-coded representations whereas neural networks learn distributed representations through training, then the conceptual relevance of comparing symbolic and deep-learning models becomes less clear. Clarifying these distinctions and explicitly situating both modeling approaches within their respective theoretical frameworks would strengthen the conceptual grounding of the paper and help readers better understand the motivation for the comparisons made between ANNs and symbolic models.

(1.2) It is also important to note that the symbolic models employed across the three experiments differ substantially in their assumptions and implementations. As a result, while it is informative to assess whether ANNs outperform a given symbolic model within a specific experiment, comparisons across experiments do not support general conclusions about whether symbolic models or ANNs better approximate human geometric shape judgments. In principle, a different or more nuanced instantiation of a symbolic model could capture human judgments equally well or even outperform ANNs. This raises questions about whether the specific symbolic models employed here provide a meaningful benchmark for assessing alignment with human geometric perception. Moreover, the manuscript offers limited discussion of the symbolic models’ results and their comparison with ANNs. The authors may therefore wish to reconsider whether including symbolic models as a baseline is informative in this context, or alternatively, to more clearly articulate the intended role and interpretive value of symbolic models within the study as suggested in the previous comment.

(1.3) One of the main findings of the manuscript is that the size of the training dataset primarily predicts the alignment between human behavior and computer vision models. However, training set size is confounded with several other factors that could likely enhance the correspondence between humans and ANNs (see Supplementary Table 1). Larger datasets are typically associated with different training objectives (e.g., supervised ImageNet vs. contrastive CLIP or self-supervised DINO/DINOv3), substantial differences in dataset identity and visual richness (e.g., ImageNet-1k vs. ImageNet-22k or large-scale web/video corpora), increases in model capacity, and variations in pretraining pipelines, optimizers, and preprocessing. As a result, the apparent advantage of larger training sets may reflect a combination of these factors rather than dataset size per se. This confound represents a substantial limitation of the study and complicates the interpretation that training set size is the primary factor of human–model alignment in geometrical shape perception. Although this issue does not admit an easy solution given the available model zoo, it calls for a more cautious interpretation of the results, stronger methodological controls (see next comment), and a more explicit discussion of the relevant confounding factors.

(1.4) Relatedly, it would be important to clarify whether the ridge regression analysis was cross-validated. If the regression was fit on the full dataset without cross-validation, the results are better interpreted as descriptive rather than predictive, and reporting standardized coefficients and uncertainty estimates may be more informative than permutation importance alone. However, I recommend using cross-validated feature-importance estimates (e.g., permutation importance on held-out folds) to reduce optimistic bias. Depending on how alignment is computed, this could be implemented via leave-one-participant-out cross-validation, or if alignment is based on an aggregate human RDM, using bootstrap/cross-validation over stimuli (e.g., split-half or k-fold over images/conditions to build human and model RDMs).

I suggest complementing the current ridge regression analysis with a hierarchical linear mixed-effects model in which the dependent variable is each model’s human–model RDM alignment (e.g., the Spearman correlation between the upper triangles of the RDMs). While this approach will not eliminate collinearity among predictors, it provides a useful robustness check to assess whether training set size remains the dominant predictor within clusters of related models and training approaches, and it quantifies how much variance is captured by group-level effects versus dataset size. In particular, mixed-effects modeling can explicitly account for clustered dependence by including random intercepts for grouping variables such as training method and architecture family (e.g., (1|training method), (1|architecture), or potentially (1 + log(training set size) | training method) and (1 + log(training set size) | architecture)). This reduces the risk that systematic differences shared by models trained under the same pipeline or stimulus set are inadvertently attributed to dataset size, as the model can allocate variance to the appropriate group-level terms rather than forcing it into the fixed effects.

(1.5) Training set size is closely correlated with the richness and diversity of the visual diet, as larger datasets typically contain more varied and semantically rich stimuli. Importantly, Conwell et al. (2024) show through controlled analyses that it is this representational richness, rather than dataset size per se, that primarily drives increased alignment between human and model representations. This constitutes a confound that can hardly be resolved using statistical tricks in the current study, and it should therefore be explicitly acknowledged and discussed by the authors.

(1.6) A particularly interesting finding of the study is that, in Experiment 3, the authors show that ANNs exhibit sensitivity to pairings between photographs and geometric drawings. However, this result is not entirely novel, and the manuscript would benefit from citing prior related work. For example, Singer et al. (2022) demonstrated that generalization to abstracted images, such as drawings, can emerge in CNNs trained on natural images.

----------------------------------------------------------------------------------------------------------------------------

2. Minor points and clarity:

(2.1) Page 2 - Paragraph 4: “We used Representational Similarity Analysis (RSA) to compare the internal representations of humans and neural networks on quadrilaterals”. This is potentially misleading, as it suggests that the human RDMs are derived from neural data. To avoid confusion, it would be clearer to specify from the outset that these are behaviorally estimated internal representations of humans (e.g., “behaviorally estimated human representations”).

(2.2) Page 3 - Paragraph 2: The authors state that the RDM was derived from participants’ “success rates” and reaction times. This terminology may be ambiguous, as cognitive scientists typically interpret “success rate” as accuracy, whereas in the AI literature it can refer to different metrics (e.g., true positive rate). To avoid confusion, it would be helpful for the authors to explicitly define what is meant by “success rate” in this context (e.g., accuracy).

(2.3) Page 3 - Paragraph 2: Related to the point above, it is unclear how the participant RDMs were estimated. Were individual participant RDMs correlated with the model RDMs, or did the authors compute a single RDM aggregated or averaged across participants? Please clarify this in the text.

(2.4) Page 4 - Paragraph 1: It is explained that to compute the ANN RDM the layer with the highest correlation was used, and that for different networks different layers were selected. To improve clarity I suggest adding a table to the supplementary materials with the specific layer used for every model.

(2.5) Page 4 - Paragraph 2: The symbolic model used in Experiment 1 is introduced as representing quadrilaterals based on their inherent geometric properties. While the model is referenced to prior work (Sablé-Meyer et al., 2021), it would be helpful to clearly enumerate all the geometric properties that were considered and how they were encoded. This clarification would aid readers in understanding how distances between stimuli were computed.

(2.6) Page 8 - Paragraph 1: The authors introduce the cumulative distraction method as an alternative to Campbell’s approach (2024), which was noted to be sensitive to outlier distractors. However, it is unclear how the cumulative distraction method addresses this limitation. In particular, extreme distractors—those with very large or very small distances—would still contribute equally when distances are averaged, potentially reintroducing the same sensitivity to outliers. Please clarify how this method mitigates the influence of outlier distractors, or otherwise justify its advantages over Campbell’s original approach.

(2.7) The authors note a potential confound between luminance and image complexity and attempt to address this by regressing out target luminance from choice time. However, another plausible confounding factor is spatial frequency: more complex images typically contain higher spatial frequency content. The authors may therefore also wish to consider regressing out spatial frequency–related measures, but results should not be affected as spatial frequency will likely be highly correlated with luminance.

(2.8) Figure 2, 3, and 4: I suggest using more dissimilar colors for panel E.

(2.9) Please clarify what the error bars represent in Figure 3C. In addition, what do the square markers in panel D denote? I assume they correspond to a combination of ANN and symbolic models; if so, this should be explicitly stated and included in the figure legend.

----------------------------------------------------------------------------------------------------------------------------

References:

-Conwell, C., Prince, J. S., Kay, K. N., Alvarez, G. A., & Konkle, T. (2024). A large-scale examination of inductive biases shaping high-level visual representation in brains and machines. Nature communications, 15(1), 9383

-Singer, J. J., Seeliger, K., Kietzmann, T. C., & Hebart, M. N. (2022). From photos to sketches-how humans and deep neural networks process objects across different levels of visual abstraction. Journal of vision, 22(2), 4-4.

-Thompson, J. A., Sheahan, H., Dumbalska, T., Sandbrink, J. D., Piazza, M., & Summerfield, C. (2024). Zero-shot counting with a dual-stream neural network model. Neuron, 112(24), 4147-4158.

Reviewer #2: This paper reports computational modelling results from three studies, in each of which representations within several deep neural networks (DNNs) are compared to previously-collected human data. The three studies involve different aspects of shape processing:

- Experiment 1: representational similarity in DNN layers is compared to human perceived similarity of simple quadrilaterals (inferred through speed and accuracy at detecting an outlying shape in a display)

- Experiment 2: representational distance in DNN layers is compared to human response time at matching a shape to that in a delayed display, using variously complex shapes

- Experiment 3: recognition (inferred through representational similarity) in DNN layers is evaluated for matching photos, line drawings, or geometric cartoons of objects, all of which can be perfectly recognised by human observers.

The results add to the literature on shape processing in DNNs, and the work is to be commended for the large number, diversity, and recency of DNNs evaluated.

MAJOR COMMENTS

The main issue with the current version of the paper is that it is missing a "Materials and Methods" section. Not enough detail to replicate the studies is provided in the main text, and the Supplementary Information contains only a few additional tables and figures. PLoS Comp Biol format generally involves a Methods section at the end of the manuscript. Such a section should be added, including the following:

- Explain software and hardware used in evaluating models and analysing data

- Explain stimuli, e.g. number of stimuli, how they were generated, resolution and colour of images, etc

- Explain model testing procedure, e.g. image preprocessing, number and method of image augmentation used to derive the "prototypical" representation for each image

- Explain all human experiments in brief - how were images presented, how long for, and how was the task phrased?

- Explain data analysis in more detail, i.e. each step involved in extracting and averaging model RDMs, each step involved in turning human response data into RDMs (e.g. in Expt 1, did behavioural RDMs somehow include both accuracy and response time data?), how model and human RDMs were compared (e.g. it seems that individual participant RDMs were averaged together, and the group-average RDM was used as the target with which to correlate each model RDM? But this is not explained explicitly.), and, in more detail, how noise ceilings were calculated .

I will likely have more methodological comments once the methods are clearer. For now, I am wondering about the reason for the prototype procedure (averaging representation over multiple image instances) used in Experiment 1? This seems likely to tilt the results in the networks' favour, by artificially creating a "representation" which abstracts away from image specifics. An argument could perhaps be made for averaging over multiple translated snapshots (to simulate human eye movements during a trial) but here the averaging is done over scale and rotation, not translation. Is this analogous to human data in some way, e.g. is the human data also averaged over multiple trials? Although this doesn't undermine the results of Experiment 1, since the same procedure was applied to all models, it may inflate human correspondence relative to other studies which generally use only a single image to measure the representation of a stimulus. The decision should at least be explained and its implications discussed.

MINOR COMMENTS

- Please ensure there are page numbers, and ideally also line numbers, to make referring to text during review easier.

- p10: Euclidean/L2 distance is used to capture stimulus similarity in DNNs, whereas Manhattan/L1 distance is used in the symbolic model. Please explain why, and whether results differ if the same distance metric is used for both.

- p10: Move paragraph containing "we systematically evaluated various vision models..." higher up in this Experiment 1 section, and explain more precisely which models were evaluated. E.g. state the number of models of each type (convolutional, ViT, supervised, unsupervised), what data they were trained on, and how they were accessed (e.g. weights of open-source pretrained networks were downloaded from original repositories).

- Table S1 caption should explain all abbreviations used in the table (e.g. state what data each training set includes and what the task is (for supervised training); define cnn and vit, define clip)

- Figure 2D - it would be helpful to label a few of the model datapoints in this plot, e.g. the best and worst performing in each class, and/or those picked out for further examination in 2E

- p14: "sum of the inverse distances" - I am not familiar with the term "inverse distance"; please explain how this differs mathematically from the sum of the distances.

- Figure 3C: Please replace the term "model difficulty" in title and axes with a more neutral description of the quantity calculated, e.g. "inter-stimuli distance" or "distance in model"

- Figure 3D: Not clear that text "Neural networks + symbolic model" floating in the middle of the plot refers to the square datapoints. Please clarify through use of legend or text positioning.

- Experiment 3 should be discussed in the context of other papers that have examined representation and recognition of line drawings in DNNs, e.g. Kubilius et al. (2016), Fan et al (2018), Singer et al (2022)

- p20: "Artificial neural networks have long been recognised....seminal work of Yamins et al (2014)". In the history of neural networks being considered as models of human visual processing, this is a very recent paper. E.g. work by Jay McLelland or Terry Sejnowski in the 1980s, or Riesenhuber & Poggio in the 1990s.

REFERENCES

Fan, J. E., Yamins, D. L. K., & Turk-Browne, N. B. (2018). Common Object Representations for Visual Production and Recognition. _Cognitive Science,_ 42(8), 2670–2698.

Kubilius, J., Bracci, S., & Beeck, Op de, H. P. (2016). Deep Neural Networks as a Computational Model for Human Shape Sensitivity. _PLoS Computational Biology,_ 12(4), e1004896.

Singer, J. J., Seeliger, K., Kietzmann, T. C., & Hebart, M. N. (2022). From photos to sketches-how humans and deep neural networks process objects across different levels of visual abstraction. _Journal of vision_, _22_(2), 4-4.

Reviewer #3: This is an interesting and potentially valuable paper that compares how humans and DNNs represent abstract geometric shapes, extending past work on this topic. In particular, they take data from three human behavioral experiments, devise “DNN analogues” of these tasks, and compare the human data to many different DNNs, varying the architecture and training data. They also compare performance to a symbolic network that was successful in a previous study.

The manuscript nicely justifies the importance of the research question and it would indeed be an interesting finding if the newer wave of DNNs can better capture the abstract visual processing exhibited by humans than a symbolic model. However, in general I find that a bit more work is needed to convincingly demonstrate this.

SPECIFIC COMMENTS:

1. It is currently impossible for the reader to understand the comparisons to the symbolic model without explaining it in more detail. In Experiment 1 it is a vector of quadrilateral properties. It isn’t clear what the “symbolic model” corresponds to in Experiment 2. This raises a number of issues both at the level of prose clarity and the analyses that were performed:

a. A more specific label than “the symbolic model” might be nice just to establish that it is in fact the same model throughout the paper; it is confusing that it is introduced in the context of quadrilaterals in Experiment 1, but is clearly more broad in Experiment 2, and coexists alongside the MDL, which is also related to a symbolic model of the shapes.

b. In computing the representational similarity of the shapes based on the symbolic model, it seems like all of the properties are weighted equally, which arguably loads the dice against the symbolic model. What if only a subset of the properties are used by humans in the task (and are sufficient to fully predict performance), with the rest only contributing noise? Especially given that these properties seem to be of different kinds (“symmetry”, “right angles”, “parallel lines”), it isn’t clear that an unweighted L1 norm makes a lot of sense. Along the same lines, I take it Pearson correlation was used to compare RDMs—this at the very least requires justification (why not use Spearman?), since human reaction times and DNN activation distances are rather different quantities.

c. To derive the “prototype patterns” for each quadrilateral, they took the average embedding across different scales and orientations. I assume this was not also done for the symbolic model because the properties in the symbolic model are already scale/rotation invariant (something else that would be nice to know). I additionally assume that this is meant to reflect the actual outlier detection task, in which the distractor shapes also varied based on their scale and orientation. While this seems fair from one perspective, one might also argue that it’s loading the dice in favor of DNNs: averaging out all the scale- and orientation-specific features will obviously increase the prominence of the abstract, invariant features in the prototype embedding, further complicating the comparison between the DNNs, the symbolic models, and the human data (and after all, this “prototype” is a statistical abstraction that doesn’t arise from any real single input to the DNN). Further justification for these analysis steps is needed.

d. This prototype-averaging procedure also complicates the later analysis where they look at how low-level visual features contribute to the results—doesn’t averaging across scales and orientations throw out many of these low-level features? Again, more justification would be nice.

2. It was not clear why these three tasks were chosen. I assume these are not the only behavioral tasks measuring the similarity of abstract shapes; if there’s particular reason for using data from these experiments over others it’d be nice for it to be spelled out.

3. A bit more clarity in the introduction would be nice, as the words “abstract” and “geometric” can be interpreted different ways. It’s eventually clear enough what the authors mean, but a few examples (akin to the intro of Experiment 3) could help the reader zero in more quickly.

4. In Figure 1 I don’t love “geometric shapes” as a subheading, since the quadrilaterals are also “geometric shapes.” Maybe something like “diverse geometric shapes”? A few more examples of the shapes might enrich this figure a bit.

5. In Experiment 1 it looks like you are using a Pearson correlation coefficient as the DV in multiple regression. Strictly speaking I believe this isn’t kosher (since Pearson correlation is bounded between -1 and +1) and violates the assumptions of linear regression. I think it’s quite plausible this won’t matter, but this needs to be addressed in some way.

6. In Figure 2, subpanels A and B are flipped.

7. In Experiment 2, you nicely regress out mean luminance as a potential confound with the complexity of the shape. But aren’t there other such confounds too (area, number of edges, etc.)? I would need more convincing that luminance is the only confound of this kind.

8. In Figure 3, the meaning of “cost” is not immediately clear and prevents the figure from being self-contained.

9. The logical relationship of Experiment 3 to the other two experiments is a bit unclear—we have lost the symbolic model that seemed key to the other two experiments and the stimuli are quite different. Again, some rationale for why these three experiments were chosen could be helpful.

10. Minor quibble, but referring to 12 years as a “long tradition” (in the Discussion section) reads a little odd.

11. The discussion is theoretically rich, but would benefit from a more thorough comparison between the symbolic model and the DNNs, as noted earlier, and the claims about a “Language of Thought” seem like a bit of a stretch from the actual findings of the paper.

**********

**Have the authors made all data and (if applicable) computational code underlying the findings in their manuscript fully available?**

The PLOS Data policy requires authors to make all data and code underlying the findings described in their manuscript fully available without restriction, with rare exception (please refer to the Data Availability Statement in the manuscript PDF file). The data and code should be provided as part of the manuscript or its supporting information, or deposited to a public repository. For example, in addition to summary statistics, the data points behind means, medians and variance measures should be available. If there are restrictions on publicly sharing data or code —e.g. participant privacy or use of data from a third party—those must be specified.

Reviewer #1: Yes

Reviewer #2: None

Reviewer #3: Yes

**********

PLOS authors have the option to publish the peer review history of their article (what does this mean?). If published, this will include your full peer review and any attached files.

If you choose “no”, your identity will remain anonymous but your review may still be made public.

**Do you want your identity to be public for this peer review?**  For information about this choice, including consent withdrawal, please see our Privacy Policy.

Reviewer #1: No

Reviewer #2: No

Reviewer #3: No

**Figure resubmission:**

While revising your submission, we strongly recommend that you use PLOS’s NAAS tool (https://ngplosjournals.pagemajik.ai/artanalysis) to test your figure files. NAAS can convert your figure files to the TIFF file type and meet basic requirements (such as print size, resolution), or provide you with a report on issues that do not meet our requirements and that NAAS cannot fix.

After uploading your figures to PLOS’s NAAS tool - https://ngplosjournals.pagemajik.ai/artanalysis, NAAS will process the files provided and display the results in the "Uploaded Files" section of the page as the processing is complete. If the uploaded figures meet our requirements (or NAAS is able to fix the files to meet our requirements), the figure will be marked as "fixed" above. If NAAS is unable to fix the files, a red "failed" label will appear above. When NAAS has confirmed that the figure files meet our requirements, please download the file via the download option, and include these NAAS processed figure files when submitting your revised manuscript.

**Reproducibility:**

To enhance the reproducibility of your results, we recommend that authors of applicable studies deposit laboratory protocols in protocols.io, where a protocol can be assigned its own identifier (DOI) such that it can be cited independently in the future. Additionally, PLOS ONE offers an option to publish peer-reviewed clinical study protocols. Read more information on sharing protocols at https://plos.org/protocols?utm_medium=editorial-email&utm_source=authorletters&utm_campaign=protocols

# Author comment: Author response to Decision Letter 1

**DOI:** 10.1371/journal.pcbi.1014554.r002

1 Apr 2026

Attachment Submitted filename: Response to reviewers.pdf

# Peer review: Decision Letter 1

**DOI:** 10.1371/journal.pcbi.1014554.r003

**Authors:** Lyle Graham, Alejandro Tabas

1 May 2026

PCOMPBIOL-D-25-02201R1

Can Neural Networks model the human perception of geometric shape

PLOS Computational Biology

Dear Dr. Pajot,

Thank you for submitting your manuscript to PLOS Computational Biology. After careful consideration, we feel that it has merit but does not fully meet PLOS Computational Biology's publication criteria as it currently stands. Therefore, we invite you to submit a revised version of the manuscript that addresses the points raised during the review process.

Please submit your revised manuscript by Jul 01 2026 11:59PM. If you will need more time than this to complete your revisions, please reply to this message or contact the journal office at ploscompbiol@plos.org. When you're ready to submit your revision, log on to https://www.editorialmanager.com/pcompbiol/ and select the 'Submissions Needing Revision' folder to locate your manuscript file.

Please include the following items when submitting your revised manuscript:

* A letter that responds to each point raised by the editor and reviewer(s). You should upload this letter as a separate file labeled 'Response to Reviewers'. This file does not need to include responses to formatting updates and technical items listed in the 'Journal Requirements' section below.

* A marked-up copy of your manuscript that highlights changes made to the original version. You should upload this as a separate file labeled 'Revised Manuscript with Track Changes'.

* An unmarked version of your revised paper without tracked changes. You should upload this as a separate file labeled 'Manuscript'.

If you would like to make changes to your financial disclosure, competing interests statement, or data availability statement, please make these updates within the submission form at the time of resubmission. Guidelines for resubmitting your figure files are available below the reviewer comments at the end of this letter

We look forward to receiving your revised manuscript.

Kind regards,

Alejandro Tabas, Ph.D.

Academic Editor

PLOS Computational Biology

Lyle Graham

Section Editor

PLOS Computational Biology

**Journal Requirements:**

1) Please ensure that the Title in your manuscript file and the Title provided in your online submission form are the same.

2) We have noticed that you have uploaded Supporting Information files, but you have not included a list of legends. Please add a full list of legends for your Supporting Information files after the references list.

**Reviewers' comments:**

Reviewer's Responses to Questions

**Comments to the Authors:**

**Please note here if the review is uploaded as an attachment.**

Reviewer #1: The review will be uploaded as an attachment.

Reviewer #2: The authors have done an impressive amount of work in this revision. They have added an extensive and informative Materials and Methods section in response to my request for one (and several other methods clarification questions from other reviewers). And they have engaged thoughtfully with the various critiques and suggestions raised in review. Methodological decisions that I found puzzling in the first submission (e.g. averaging DNN activations over multiple presentations of a stimulus) are well justified now that further detail has been provided about the human experimental design. I find the revised paper interesting and well presented and have no further comments.

Reviewer #3: I thank the authors for their thoughtful changes to the manuscript in response to reviewer comments. The added methods section and details about the symbolic models have greatly improved clarity, and the supplementary statistical analyses (e.g., using spearman instead of Pearson; applying Fisher Z-transform to the correlation values prior to the regression analysis; controlling for low-level factors in addition to luminance) have done much to shore up the sturdiness of these findings. Additionally, the prose-level changes have nicely improved the clarity of the manuscript.

That said, there remains one major issue I would like to see addressed.

Both I and another reviewer raised the point that averaging the DNN embeddings across variation in scale and angle in Experiment 1 might artificially tilt the scales towards finding evidence of “symbolic” representations in DNNs. The authors responded that the human data is also aggregated across variation in scale and angle, so this averaging step remains valid.

However, this response elides a subtle but crucial distinction. In the human case, shapes with a particular size/orientation are presented, and the time/accuracy for finding the “odd one out” is measured. These behavioral measures—which constitute the measured dissimilarity for the purposes of RSA—are collected in an experimental setting that includes size/orientation variation, and are only subsequently averaged.

By contrast, for the DNNs the embeddings for different sizes/orientations are averaged first, and the dissimilarity is computed afterward.

These forms of averaging are not the same: for humans dissimilarity is measured between specific exemplars of each shape and averaged afterwards (i.e., average distance between specific exemplars), and for DNNs dissimilarity is computed between constructed statistical “prototypes” that don’t correspond directly to any actual input seen by the DNN (i.e., distance between average of exemplars). The truly analogous procedure would be: for shapes A and B, feed all size/orientation variants of A and B into the DNN, compute the distance between each variant of A and each variant of B (taking all combinations), and only then take the average distance. It is easy to see that these procedures will yield different measured dissimilarities: given two multivariate Gaussians, the distance between random draws of those Gaussians will in general be bigger than the distance between their centroids. Furthermore, this distinction is of theoretical interest: it could be the case that DNNs contain size/orientation invariant visual information (as they almost surely do), but that this information plays a smaller role in the representation than the “nuisance” variables that are discarded in the averaging procedure.

The authors are correct that they apply the same procedure to all DNNs, so this issue doesn’t apply to the between-DNN comparisons, but since the comparison between DNNs and symbolic models is foundational to the manuscript, this point simply must be addressed to warrant the authors’ comparative claims.

**********

**Have the authors made all data and (if applicable) computational code underlying the findings in their manuscript fully available?**

The PLOS Data policy requires authors to make all data and code underlying the findings described in their manuscript fully available without restriction, with rare exception (please refer to the Data Availability Statement in the manuscript PDF file). The data and code should be provided as part of the manuscript or its supporting information, or deposited to a public repository. For example, in addition to summary statistics, the data points behind means, medians and variance measures should be available. If there are restrictions on publicly sharing data or code —e.g. participant privacy or use of data from a third party—those must be specified.

Reviewer #1: **No:** Despite the addition of a Methods section, the authors have not included a statement regarding data and code availability, which I have now raised as a major concern. Additionally, key aspects of the Methods remain insufficiently described and would benefit from further refinement to ensure the transparency and reproducibility of the results.

Reviewer #2: Yes

Reviewer #3: Yes

**********

PLOS authors have the option to publish the peer review history of their article (what does this mean?). If published, this will include your full peer review and any attached files.

If you choose “no”, your identity will remain anonymous but your review may still be made public.

**Do you want your identity to be public for this peer review?**  For information about this choice, including consent withdrawal, please see our Privacy Policy.

Reviewer #1: No

Reviewer #2: No

Reviewer #3: No

[NOTE: If reviewer comments were submitted as an attachment file, they will be attached to this email and accessible via the submission site. Please log into your account, locate the manuscript record, and check for the action link "View Attachments". If this link does not appear, there are no attachment files.]

**Figure resubmission:**

While revising your submission, we strongly recommend that you use PLOS’s NAAS tool (https://ngplosjournals.pagemajik.ai/artanalysis) to test your figure files. NAAS can convert your figure files to the TIFF file type and meet basic requirements (such as print size, resolution), or provide you with a report on issues that do not meet our requirements and that NAAS cannot fix.

After uploading your figures to PLOS’s NAAS tool - https://ngplosjournals.pagemajik.ai/artanalysis, NAAS will process the files provided and display the results in the "Uploaded Files" section of the page as the processing is complete. If the uploaded figures meet our requirements (or NAAS is able to fix the files to meet our requirements), the figure will be marked as "fixed" above. If NAAS is unable to fix the files, a red "failed" label will appear above. When NAAS has confirmed that the figure files meet our requirements, please download the file via the download option, and include these NAAS processed figure files when submitting your revised manuscript.

**Reproducibility:**

To enhance the reproducibility of your results, we recommend that authors of applicable studies deposit laboratory protocols in protocols.io, where a protocol can be assigned its own identifier (DOI) such that it can be cited independently in the future. Additionally, PLOS ONE offers an option to publish peer-reviewed clinical study protocols. Read more information on sharing protocols at https://plos.org/protocols?utm_medium=editorial-email&utm_source=authorletters&utm_campaign=protocols

Attachment Submitted filename: PLOS_CB_round2.pdf

# Author comment: Author response to Decision Letter 2

**DOI:** 10.1371/journal.pcbi.1014554.r004

9 Jun 2026

Attachment Submitted filename: Response to reviewers.docx

# Peer review: Decision Letter 2

**DOI:** 10.1371/journal.pcbi.1014554.r005

**Authors:** Lyle Graham, Alejandro Tabas

3 Jul 2026

PCOMPBIOL-D-25-02201R2

Can Neural Networks model the human perception of geometric shapes?

PLOS Computational Biology

Dear Dr. Pajot,

Thank you for submitting your manuscript to PLOS Computational Biology. After careful consideration, we feel that it has merit but does not fully meet PLOS Computational Biology's publication criteria as it currently stands. Therefore, we invite you to submit a revised version of the manuscript that addresses the points raised during the review process.

Please submit your revised manuscript by Sep 02 2026 11:59PM. If you will need more time than this to complete your revisions, please reply to this message or contact the journal office at ploscompbiol@plos.org. When you're ready to submit your revision, log on to https://www.editorialmanager.com/pcompbiol/ and select the 'Submissions Needing Revision' folder to locate your manuscript file.

Please include the following items when submitting your revised manuscript:

* A letter that responds to each point raised by the editor and reviewer(s). You should upload this letter as a separate file labeled 'Response to Reviewers'. This file does not need to include responses to formatting updates and technical items listed in the 'Journal Requirements' section below.

* A marked-up copy of your manuscript that highlights changes made to the original version. You should upload this as a separate file labeled 'Revised Manuscript with Track Changes'.

* An unmarked version of your revised paper without tracked changes. You should upload this as a separate file labeled 'Manuscript'.

If you would like to make changes to your financial disclosure, competing interests statement, or data availability statement, please make these updates within the submission form at the time of resubmission. Guidelines for resubmitting your figure files are available below the reviewer comments at the end of this letter.

As the corresponding author, your ORCID iD is verified in the submission system and will appear in the published article. PLOS supports the use of ORCID, and we encourage all coauthors to register for an ORCID iD and use it as well. Please encourage your coauthors to verify their ORCID iD within the submission system before final acceptance, as unverified ORCID iDs will not appear in the published article. Only the individual author can complete the verification step; PLOS staff cannot verify ORCID iDs on behalf of authors.

We look forward to receiving your revised manuscript.

Kind regards,

Alejandro Tabas, Ph.D.

Academic Editor

PLOS Computational Biology

Lyle Graham

Section Editor

PLOS Computational Biology

**Additional Editor Comments (if provided):**

I would like to ask you to please consider the last open points of Reviewer 1, specially the ones referring to Figure 1, the noise ceilings, and the abstract. Please feel free to implement the other suggestions only if you consider they will improve the manuscript. We will be pleased to accept the paper for publication without any further round of reviews after we have received the revised manuscript.

**Journal Requirements:**

If the reviewer comments include a recommendation to cite specific previously published works, please review and evaluate these publications to determine whether they are relevant and should be cited. There is no requirement to cite these works unless the editor has indicated otherwise.

1) We have noticed that you have uploaded Supporting Information files, but you have not included a list of legends. Please add a full list of legends for your Supporting Information files after the references list.

**Reviewers' comments:**

Reviewer's Responses to Questions

**Comments to the Authors:**

**Please note here if the review is uploaded as an attachment.**

Reviewer #1: I would like to thank the authors for the considerable effort they have invested throughout the review process. The revisions have substantially improved the manuscript by clarifying its objectives and scope, providing a more detailed and transparent description of the methods, and offering a more thorough and nuanced discussion of the findings. In my view, these changes have not only improved the readability of the paper but have also strengthened the interpretability and significance of the results, thereby broadening the manuscript’s appeal to a wider readership. I am therefore pleased to recommend the manuscript for publication, contingent upon the authors addressing the minor points outlined below.

1. Introduction:

-Page 1, second paragraph: The authors begin with the phrase “In line with the Language of Thought Hypothesis”, but the hypothesis has not yet been introduced. It would be preferable to first define the Language of Thought Hypothesis and then refer back to it.

-Page 1, third paragraph: The manuscript refers to “classicist theories of cognition”, but this term is not clearly defined. I would personally avoid using the term “classicist”, because given the long history of connectionist models, some readers may also view them as “classical” theories in a broader sense.

-Page 2: While I understand the authors’ intention, the following sentence reads somewhat awkwardly: “We do not try to adjudicate between symbolic and connectionist accounts of geometric cognition, and pursue a more tractable goal.” Because the second clause contrasts with the first rather than simply adding information, a construction using a contrastive expression (e.g., “instead” or “rather than”) may better convey the intended meaning than the copulative conjunction “and”.

-The Introduction states twice that the symbolic models of Sablé-Meyer et al. (2021, 2022) are used as baselines. This point only needs to be made once and could be streamlined.

2. Figure 1: The authors indicate that the image representing the symbolic model has been modified; however, the figure appears unchanged from the previous version. In addition, Figure 1 currently lacks a descriptive caption header.

3. Noise ceilings: I understand the authors’ rationale for their approach to estimating noise ceilings, although it may be considered somewhat unorthodox. I do not intend to revisit this issue in a third round of review. Nevertheless, given the central importance of noise ceilings in a correlational study of this kind, I believe the authors should explicitly acknowledge the relatively low between-subject reliability of the behavioral data as a limitation of the current study in the Discussion.

4. Abstract: The authors refer to a “language of geometry” in the Abstract, but this term does not appear elsewhere in the manuscript. To avoid potential confusion, I would suggest either removing the term from the Abstract or introducing and defining it in the Introduction. If retained, it may fit naturally alongside the introduction of the Language of Thought hypothesis (see comment above).

Reviewer #3: The authors have suitably addressed my remaining concerns, and I congratulate them on this rich and informative study.

**********

**Have the authors made all data and (if applicable) computational code underlying the findings in their manuscript fully available?**

The PLOS Data policy requires authors to make all data and code underlying the findings described in their manuscript fully available without restriction, with rare exception (please refer to the Data Availability Statement in the manuscript PDF file). The data and code should be provided as part of the manuscript or its supporting information, or deposited to a public repository. For example, in addition to summary statistics, the data points behind means, medians and variance measures should be available. If there are restrictions on publicly sharing data or code —e.g. participant privacy or use of data from a third party—those must be specified.

Reviewer #1: Yes

Reviewer #3: Yes

**********

PLOS authors have the option to publish the peer review history of their article (what does this mean?). If published, this will include your full peer review and any attached files.

If you choose “no”, your identity will remain anonymous but your review may still be made public.

**Do you want your identity to be public for this peer review?**  For information about this choice, including consent withdrawal, please see our Privacy Policy.

Reviewer #1: No

Reviewer #3: No

[NOTE: If reviewer comments were submitted as an attachment file, they will be attached to this email and accessible via the submission site. Please log into your account, locate the manuscript record, and check for the action link "View Attachments". If this link does not appear, there are no attachment files.]

**Figure resubmission:**

-->While revising your submission, we strongly recommend that you use PLOS’s NAAS tool (https://ngplosjournals.pagemajik.ai/artanalysis) to test your figure files. NAAS can convert your figure files to the TIFF file type and meet basic requirements (such as print size, resolution), or provide you with a report on issues that do not meet our requirements and that NAAS cannot fix.

After uploading your figures to PLOS’s NAAS tool - https://ngplosjournals.pagemajik.ai/artanalysis, NAAS will process the files provided and display the results in the "Uploaded Files" section of the page as the processing is complete. If the uploaded figures meet our requirements (or NAAS is able to fix the files to meet our requirements), the figure will be marked as "fixed" above. If NAAS is unable to fix the files, a red "failed" label will appear above. When NAAS has confirmed that the figure files meet our requirements, please download the file via the download option, and include these NAAS processed figure files when submitting your revised manuscript.

**Reproducibility:**

To enhance the reproducibility of your results, we recommend that authors of applicable studies deposit laboratory protocols in protocols.io, where a protocol can be assigned its own identifier (DOI) such that it can be cited independently in the future. Additionally, PLOS ONE offers an option to publish peer-reviewed clinical study protocols. Read more information on sharing protocols at https://plos.org/protocols?utm_medium=editorial-email&utm_source=authorletters&utm_campaign=protocols

# Author comment: Author response to Decision Letter 3

**DOI:** 10.1371/journal.pcbi.1014554.r006

7 Jul 2026

Attachment Submitted filename: Reviewer responses.docx

# Editor report: Decision Letter 3

**DOI:** 10.1371/journal.pcbi.1014554.r007

**Authors:** Lyle Graham, Alejandro Tabas

9 Jul 2026

Dear Mr Pajot,

We are pleased to inform you that your manuscript 'Can Neural Networks model the human perception of geometric shapes?' has been provisionally accepted for publication in PLOS Computational Biology.

Before your manuscript can be formally accepted you will need to complete some formatting changes, which you will receive in a follow up email. A member of our team will be in touch with a set of requests.

Please note that your manuscript will not be scheduled for publication until you have made the required changes, so a swift response is appreciated.

IMPORTANT: The editorial review process is now complete. PLOS will only permit corrections to spelling, formatting or significant scientific errors from this point onwards. Requests for major changes, or any which affect the scientific understanding of your work, will cause delays to the publication date of your manuscript.

Should you, your institution's press office or the journal office choose to press release your paper, you will automatically be opted out of early publication. We ask that you notify us now if you or your institution is planning to press release the article. All press must be co-ordinated with PLOS.

Thank you again for supporting Open Access publishing; we are looking forward to publishing your work in PLOS Computational Biology.

Best regards,

Alejandro Tabas, Ph.D.

Academic Editor

PLOS Computational Biology

Lyle Graham

Section Editor

PLOS Computational Biology

***********************************************************

# Editor report: Acceptance letter

**DOI:** 10.1371/journal.pcbi.1014554.r008

**Authors:** Lyle Graham, Alejandro Tabas

PCOMPBIOL-D-25-02201R3

Can Neural Networks model the human perception of geometric shapes?

Dear Dr Pajot,

I am pleased to inform you that your manuscript has been formally accepted for publication in PLOS Computational Biology. Your manuscript is now with our production department and you will be notified of the publication date in due course.

The corresponding author will soon be receiving a typeset proof for review, to ensure errors have not been introduced during production. Please review the PDF proof of your manuscript carefully, as this is the last chance to correct any errors. Please note that major changes, or those which affect the scientific understanding of the work, will likely cause delays to the publication date of your manuscript.

Soon after your final files are uploaded, unless you have opted out, the early version of your manuscript will be published online. The date of the early version will be your article's publication date. The final article will be published to the same URL, and all versions of the paper will be accessible to readers.

For Research, Software, and Methods articles, you will receive an invoice from PLOS for your publication fee after your manuscript has reached the completed accept phase. If you receive an email requesting payment before acceptance or for any other service, this may be a phishing scheme. Learn how to identify phishing emails and protect your accounts at https://explore.plos.org/phishing.

Thank you again for supporting PLOS Computational Biology and open-access publishing. We are looking forward to publishing your work!

With kind regards,

Janani Seenivasan

PLOS Computational Biology | Carlyle House, Carlyle Road, Cambridge CB4 3DN | United Kingdom ploscompbiol@plos.org | Phone +44 (0) 1223-442824 | ploscompbiol.org | @PLOSCompBiol
